# Validation and psychometric properties of the Russian version of the Touch Experiences and Attitudes Questionnaire (TEAQ-37 Rus)

**DOI:** 10.1371/journal.pone.0206905

**Published:** 2018-12-13

**Authors:** Paula Trotter, Elena Belovol, Francis McGlone, Anton Varlamov

**Affiliations:** 1 Natural Sciences and Psychology, Liverpool John Moores University, Liverpool, United Kingdom; 2 Center for Cognition and Communication, Pushkin State Russian Language Institute, Moscow, Russia; 3 Department of Psychology, Moscow State University of Education, Moscow, Russia; 4 Institute of Psychology, Health & Society, University of Liverpool, Liverpool, United Kingdom; 5 Laboratory of Psychophysiology, Institute of Higher Nervous Activity and Neurophysiology of RAS, Moscow, Russia; Universitetet i Oslo, NORWAY

## Abstract

It has been demonstrated that nurturing and affiliative touch is essential for human emotional and physical well-being throughout our entire life. Within the last 30 years a system of low-threshold mechanosensitive C fibers innervating the hairy skin was discovered and described; this system is hypothesized to represent the neurobiological substrate for the affective and rewarding properties of touch. This discovery opens new perspectives for multidisciplinary research of the role of affiliative social touch in health and disease, and calls for establishing novel psychometric tools assessing individual differences in the domain of affective touch. The main objective of the study was to construct and validate a Russian version of the Touch Experiences and Attitudes Questionnaire (TEAQ), a self-report measure recently developed to quantify individual experience and attitude to social and affective touch. A pool of 117 items was translated into Russian and all the items were assessed for appropriateness for Russian culture (232 participants). After exploring the factor structure (468 participants), we composed a 37-item questionnaire (TEAQ-37 Rus) characterized by good reliability and a clear 5-factor structure, covering the aspects of attitude to intimate touch, attitude to friendly touch, attitude to self-care, current intimate touch experiences, and childhood touch experiences. Confirmatory factor analysis (551 participants) has demonstrated good consistency and reliability of the 5-factor structure of the TEAQ-37 Rus. Cross-validation research demonstrated moderate positive correlations between predisposition to social touch and emotional intelligence; positive correlations with extraversion and openness facets of the Big Five personality model were also found. As predicted, participants with higher TEAQ-37 Rus scores rated all observed kinds of touch as more pleasant, with a particular preference for slow touch. We anticipate that this questionnaire will be a valuable tool for researchers of social touch, touch perception abnormalities, and the importance of touch experiences for emotional and mental health.

## Introduction

### Affective touch throughout human life

Communication via the sense of touch has long been perceived as an important aspect of human social interaction. A large body of literature attests to its cultural, social, and emotional significance and it may seem natural to acknowledge the importance of gentle caring touch and the role it plays in our social and emotional well-being, but there was no general agreement about this amongst psychologists up until the mid 20^th^ century. John B. Watson, an instigator of the School of Behaviorism and one of the most influential psychologists of early 20^th^ century, stated that, in order to bring up their children properly, parents should “never hug and kiss them, never let them sit on your lap”. An untouched child would “enter manhood so bulwarked with stable work and emotional habits that no adversity can quite overwhelm him” [1]. His approach was shared by Haarer [2], who authored one of the most popular German books on child care for several decades, with the last edition published as late as 1987 [3]. A similar point of view, if not as radical, is still popular in some cultures, and parents are often advised not to ‘spoil’ their children with excessive physical affection [4]. In the 1940’s and 1950’s revolutionary research carried out by Spitz in nurseries and infant hospitals [5] proved that a generous amount of nurturing touch is as vital as air and food, and that infants devoid of caring touch often die from a so-called ‘hospitalism’, a condition described in late 19^th^ century referring to infants’ failure to thrive and to stunningly high death rates [6]. Impressed by Spitz’s work, Berne postulates that “a stroke may be used as the fundamental unit of social action” [7]. A mother’s reassuring touch is linked to a more beneficial type of attachment in view of Bowlby’s theory [8]: a securely attached infant both seeks and is comforted by physical contact with their mother [9]; a comprehensive review of the data linking touch and attachment is provided by Duhn [10]. The importance of touch for shaping the emotional brain is thoroughly supported by animal research data. A classical paper by Harlow [11] shows that infant monkeys who had been removed from their mothers prefer a surrogate mother made of soft cloth to one made of wire that provided food, pinpointing the importance of tactile perception in nurturing. The work of Meaney [12] provided further evidence that rat pups receiving high levels of licking and grooming touch in the early neonatal period have significantly lower stress responses, an effect which prevails to adulthood: adult offsprings with increased licking-grooming show lower responses to stress [13]. Recently this protecting effect of maternal touch has been replicated in humans: a copious amount of maternal stroking can reverse the potentially harmful epigenetic effects induced by prenatal maternal depression followed by postnatal maternal depression [14].

Affective touch retains its key role for human emotional well-being throughout our entire life. Cochrane [15] identified that a lack of social touch, either during childhood or at present, greatly increased one’s vulnerability to depression. Eaton et al. found that a simple touch on the shoulder before mealtime resulted in an increase of nutritional intake in institutionalized elderly, preventing health risks related to malnutrition [16]. Further evidence of the benefits of touch comes from research on the effects of massage showing a reduction in salivary cortisol, an increase in urinary serotonin metabolite levels, and reduction in depression and pain [17]. The popularity of massage in improving well-being is known in many cultures, and there is a plethora of less founded ‘alternative medicine’ based therapies claiming miracle “cures” as a consequence of the laying-on of hands. However, until recently, a neurobiological explanation of these benefits has been lacking with most research in the area being carried out by psychologists, ethologists and social care professionals.

### C-tactile system: Neural substrate mediating affective touch perception

Neurobiological research performed within the last 25 years has reinforced the earlier behavioral insights into the importance of touch for child’s development and revealed that there indeed is a specific neural substrate for perceiving the emotional properties of gentle touch. Our current understanding is that the human somatosensory system has in fact two tactile sub-modalities, one providing the well-recognized discriminative touch input to the brain, and the second–the affective or emotional input. A system of low-threshold mechanosensitive C-fibers innervating the hairy skin of the body (C-tactile or CT-afferents) has been identified and characterized [18–20]; this system is hypothesized to represent the neurobiological substrate for affective and rewarding properties of touch (for review see [21]). These nerve fibers are slowly conducting and respond to low-force, innocuous touch; they were first discovered by Vallbo et al. [18] using a technique called microneurography that allows electrophysiological recording of the activity of single axons in a conscious participant [22]. Electrophysiological and psychophysical research revealed that properties of CT fibers and the corresponding mechanoreceptors are optimized for response to naturally occurring nurturing touch, i.e. to stroking stimuli with delivered with velocity of ~5 cm/sec [20, 23] and at normal human skin temperature [24]. It has been shown that pleasant touch delivered to hairy skin is processed primarily in limbic-related cortex [25–27, 20]. The CT system, with its slow response to stimulation and lack of topographic specificity, is best equipped to fulfill and affective rather than discriminative function, encoding the rewarding and affiliative properties of close physical contact. It provides positive reinforcement to skin-to skin contacts with other people, serves as a reward mechanism enhancing attachment, and helps to keep us ‘in touch’, both literally and figuratively. The CT affective touch hypothesis is presented in authoritative review papers [28, 29] and in major textbooks of neuroscience [30–34].

### Assessing affective touch

The majority of papers revealing the link between CT system, social touch, and neurodevelopment were published within the last three decades, and it is becoming clear that this area of research is crucial for understanding neural mechanisms underlying different aspects of human somatosensory perception and can be vital in research on a range of developmental, neurological, and behavioral disorders related to tactile perception abnormalities.

The main factors affecting touch experience and attitudes can be grouped into two clusters: 1) physical properties of a delivered stimulus (force, velocity, texture, temperature etc.), along with the properties or conditions of the skin being touched, and 2) the factors related to social and cultural context. Probably the most important social factor regulating permissibility of social touch and influencing touch-related emotional experience is the strength of the social bond between the interacting people [33]. According to the touch attitudes and behaviors prevailing in a given culture, a culture can be classified as contact or non-contact [34]. Typical interpersonal touch patterns may vary widely depending on social bond strength (partners, relatives, friends, strangers) or for different contexts related to age, gender, social roles etc. It has also been demonstrated that social and cultural attitudes and expectations can mediate touch perception through cognitive labelling [35] or even by feeding false information on the gender of a person providing manual touch stimulation [36]. Exposure to everyday social touch also modulates pleasantness ratings and hedonic discrimination ability [37].

To move further into the domain of translational research we have to be equipped with a range of appropriate research tools, including neuroscience methods assessing physiological responses directly, psychophysical protocols for controlled stimulus delivery, and psychometric tools and clinical scales enabling us assess behavior, attitudes, and experiences, and to take into account social and cultural factors.

#### Psychophysical protocols and stimulus databases

Robotic tactile stimulation technique (RTS) was developed to deliver stroking stimuli with maximum precision and control over timing, force and velocity [23], and several studies have used a range of experimental observation protocols using RTS [20, 23, 38, 39]. Manual stimulus delivery protocols were also used in several research papers [40–42], and it was confirmed that pleasantness rating for strokes delivered by robotic and manual stimulation correspond very closely [43]. Most of the data that laid the foundation of CT affective touch hypothesis were obtained using RTS or manual touch delivery protocols, microneurography, neuroimaging methods, and subjective rating scales (Likert type or visual analogue scales). Another approach to assess perceived pleasantness of touch was recently suggested by Walker et al. [44], who used a series of short (5 sec) video clips depicting slow and fast strokes and static touch delivered by hand to different body sites. The clips were intentionally made as impersonal as possible by choosing close up angles not revealing the faces of the actors; the somewhat artificial nature of the interaction and a clear lack of social context helps the viewers to concentrate on purely sensory aspects of touch. Subjective ratings of the perceived pleasantness of the touch were found to be very consistent and confirm that people strongly prefer slow touch to fast or static touch. A different approach was taken by Masson and Op de Beeck [45] who created and validated a set of short video clips depicting socio-affective touch events naturally occurring during different typical social contexts; this video set is more suitable for capturing the social aspects of emotional touch perception.

#### Social touch questionnaires

There is a range of scales and questionnaires assessing individual, social, and cultural differences in terms of experiences and attitudes to affiliative social touch in different situations and contexts.

Most of the available measures are related to touch perception abnormalities in childhood (for review see [32]). For the purposes of our study the most closely related questionnaires are the touch avoidance measure (TAM) [46] measuring negative attitude to touch with the opposite or same sex; the familial touch orientation scale [47] assessing familial touch experience and linking it to attitude to and frequencies of sex-related social touch in public places; its modified version, Recollection of Early Childhood Touch scale [48]; the tactile type questionnaire (TACTYPE) [49] assessing ‘tactile tendency’ (attitudes to tactile interactions with same sex or different sex peers) in college-age students; the Questionnaire on Physical Contact Experience (QPCE) [15], a very brief 8-item measure assessing experiences of good, bad, and neutral touch, currently and in childhood along with current and childhood experience of love; and the Social Touch Questionnaire [50], a 20-item scale focused on being comfortable or having negative feelings in different situations related to social touch and devised to measure the impact of social anxiety on attitude to social touch. A recently developed questionnaire, the Touch Experiences and Attitudes Questionnaire (TEAQ) [51] is, probably, the first questionnaire assessing both attitudes and life experiences that has an established and validated factor structure. The original English (UK validated) version has 57 items and includes six subscales: Friends and Family Touch (FFT), Current Intimate Touch (CIT), Childhood Touch (ChT), Attitude to Self-Care (ASC), Attitude to Intimate Touch (AIT), and Attitude to Unfamiliar Touch (AUT). The original TEAQ and the scoring instructions are provided in Supporting information (S1 Table). The validation studies ascertained its good internal consistency, construct validity in terms of discriminant validity, known-group validity and convergent validity, and criterion-related validity in terms of predictive validity and concurrent validity. Good concurrent and predictive validity of the TEAQ compared to other physical touch measures (TAM, the Familial Touch Orientation (FTO) scale, the TACTYPE questionnaire, the Touch Test, the QPCE, the Physical Contact Assessment Questionnaire and the STQ) was identified.

As for the situation in Russia, we were unable to find in Russian any psychometric measure assessing attitudes to and experiences of social touch, with a reported factor structure and psychometric properties.

### Aim and general design of the study

Our general research aim was to construct and validate a Russian version of the Touch Experiences and Attitudes Questionnaires (TEAQ). This measure would be able to assess attitudes to different kinds of social touch occurring in different social contexts, and to report childhood and current touch experiences. There are clear cultural differences in behaviors related to social touch within different cultures [52, 53], leading to possible natural differences in factor structures of different national versions of multi-factor psychometric tools. Our goal was to maximize the content validity for the Russian version, rather than mechanistically reproducing the factor structure of the original English version of the TEAQ. This was to be achieved by using a relatively wide initial pool of items (same as for the original English version of the TEAQ) and by following the same steps as in the original English study to create an operational Russian version. Such an approach may help to achieve higher content validity for each culture, similarly to the approach suggested by the creators of International Personality Item Pool [54]. Such questionnaire should also be well-suited for use with large and diverse samples of Russian-speaking respondents, including clinical and vulnerable populations, therefore special attention should be paid to good cultural admissibility of all the items. According to the aforementioned methodological considerations, the study was performed in four stages:

**Study 1:** Assessing appropriateness of the items from the original English item pool for Russian culture.
**Study 2:** Exploratory factor analysis yielding an operational Russian version of the TEAQ (the TEAQ-Rus) with acceptable consistency and reasonable factor structure.
**Study 3:** Confirming the factor structure with an independent sample of participants and reporting general psychometric properties on the TEAQ-Rus.
**Study 4:** Identifying possible demographic differences in the TEAQ-Rus responses and cross-validating the TEAQ-Rus against other personality constructs (Big Five traits and emotional intelligence) and other touch assessment tools.

In the present study we tested the following hypotheses:

1. the resulting Russian version of the TEAQ would have a consistent factor structure reflecting attitudes to social touch, and childhood and current experiences of social touch; the factor structures of English and Russian versions of the TEAQ should be reasonably similar with possible minor differences due to cultural specifics;
2. similarly to the original English version [51], the TEAQ-Rus subscale scores would be significantly influenced by gender and cohabiting conditions and, to a much smaller degree, may be influenced by education or age cohorts;
3. the subscales of the TEAQ-Rus would have good discriminant validity against other personality measures, and would show positive correlations with emotional intelligence, reflecting the affiliative role of affective touch;
4. participants with higher total TEAQ score would rate all kinds of observed touch as more pleasant, and would show stronger preference for slow strokes.

## Study 1

The aim of Study 1 was to pilot test the original pool of 117 TEAQ items, and to assess the appropriateness of the items for Russian culture and their perceived connotations. As a result of Study 1, a subset of items characterized by both adequate cultural appropriateness and reasonably high item-total correlations would be selected for further analyses.

### 1.1. Methods

#### Participants

Participants were recruited through snowball sampling. To increase control over snowball sampling, the number of the referrals was limited, all the referrals were instructed to try to collect the data from people with different age, social, and educational background, and collected responses from no more than 10 participants per referral. All the referrals were qualified psychologists (at least a BA degree in psychology); they were instructed to invite for participation people of diverse age and social backgrounds. All the participants (N = 232) freely agreed to answer a questionnaire and gave informed consent. Study 1, as with all the other Studies reported in the present article, was approved by the Pushkin Institute research ethics committee. Participants age varied between 16 and 79 years (M = 26.9, SD = 9.7), 149 participants were female (64%) and 83 (36%) were male. Male and female samples did not differ significantly in terms of age (p = 0.670); mean age and SDs were also similar (Female: Mean = 26.68, SD = 9.25; Male: Mean = 27.25, SD = 9.27).

#### Materials

The original item pool was developed in English by Trotter et al. [55] and consisted of 117 statements describing different kinds of positive affective touch (mostly hugs, kisses, skin-to-skin and hair-to-skin contact, self-care, touching animals and different textures) occurring in appropriate social contexts with partners, friends or relatives, and unfamiliar people, along with several general statements regarding social touch.

Translation of the items into Russian was performed independently by three certified translators (one holding PhD degree in Psychology, one in Neuroscience). A consensus version was composed collegially by the translators and an impartial editor. Back-translation performed independently by two translators unfamiliar to the original revealed no meaningful disagreement with the original version. The expert committee has reviewed the translation and the general suitability of the item pool (how representative are the items of Russian typical touch behaviors, how fully they cover different contexts typical for social touch in Russian culture) and has assessed both as good. The items were used with a 5-point Likert scale of agreement (‘Disagree strongly’ = 1, ‘Disagree a little’ = 2, ‘Neither agree nor disagree’ = 3, ‘Agree a little’ = 4, ‘Agree strongly’ = 5), as was suggested by the authors of the original item pool. The complete set of questions in English and in Russian is provided in Supporting information (S2 Table).

#### Procedure

The data were collected by the researchers via a paper and pencil questionnaire at a room at the university. At the beginning, the participants were told that the aim of the study was to adapt for Russian-speaking population a questionnaire originally composed in English. The participants were encouraged to make their comments regarding the content of the items, their acceptability and admissibility for Russian culture. After completion they were asked whether or not the questionnaire and individual items measures touch experiences and their attitudes to social touch, in order to assess face validity of the questionnaire. It was highlighted that there were no right or wrong answers for the items. The participants were assured that all collected data would be confidential and anonymous and that no individual data would be analyzed.

#### Qualitative and statistical analysis

For Study 1 and for all other Studies responses for negatively phrased items were reverse scored so that all item scores would reflect more positive attitude to touch or more frequent experiences. All statistical analyses were performed using Statistica 10.0 software. More than 40% percent of the participants expressed explicit complaints that the questionnaire was too long and incorporated inappropriate or seemingly irrelevant questions. According to this feedback, two simple criteria were formulated to exclude the items from the item pool used for exploratory factor analyses in Study 2:

1. Items deemed inappropriate by at least 20% of the participants were to be excluded.
2. Any items with very low item-total correlation (*r* <0.1) were to be excluded to further reduce the volume of the item pool. This low threshold was selected as we could have expected the subscales within the scale to be relatively independent from each other.

### 1.2. Results

Items containing explicit questions on intimate life were excluded as inappropriate, as 68% of participants of the Study 1 sample considered them to be inadmissible for wide use in a questionnaire for Russian culture (e.g., Q30, “I enjoy the physical intimacy of sexual foreplay”; Q57, “I enjoy having sex”). The inclusion of explicitly sex-related items that are considered inappropriate by a large part of the respondents would affect the respondents’ experiences, causing possible vexation or embarrassment of the respondents and could have compromised the integrity of respondents’ answers to the other questions.

Cronbach’s α as a measure of the TEAQ-117 internal consistency was 0.93, demonstrating a high level of items’ consistency. Despite that, 27 items had item-total correlations below 0.1; these items were also excluded from further analyses. A pilot exploratory factor analysis confirmed that none of these items had factor loadings higher than 0.4 on any of the factors for 5-factor or 6-factor models prompted by Cattell’s scree test [56]. Individual examination of items excluded due to low item-total correlations revealed that at this stage all the items concerning touch other than interpersonal touch and self-care touch were excluded, namely, touching or feeling different surfaces, “I don't like the feel of wool against my skin”, *r* = -0.04), itching (Q1, “Having an itch scratched is very enjoyable”, *r* = 0.03) along with several general items that do not relate directly to touch, concerning emotional experiences (Q62, “I was alone a lot during my childhood”, *r* = 0.00), or skin quality (Q82, “I have dry skin”, *r* = -0.04).

A pool of 85 retained items was selected for use in Study 2; each of the items was deemed appropriate for general Russian adult population.

## Study 2

The goal of the second Study was to perform exploratory analysis for the reduced 85-item Russian TEAQ pool and to construct a reasonably brief questionnaire with good content and construct validity and a consistent factor structure to serve further as a suggested operational Russian version of the TEAQ.

### 2.1. Methods

#### Participants

A separate sample of 468 participants was recruited through a highly controlled version of snowball sampling, according to the procedure described in Participants section of Study 1. All the participants freely agreed to answer a questionnaire at this stage, 306 (65%) were female and 162 (35%) were male. Participants age varied between 16 and 79 years (M = 25.9, SD = 9.7). Male and female samples did not differ significantly in terms of age (p = 0.119); mean age and SDs were also similar (Female: Mean = 25.40, SD = 9.73; Male: Mean = 26.87, SD = 9.56).

#### Materials and procedure

The participants completed a questionnaire composed of 85 TEAQ items. Data were collected personally by the researchers via a paper and pencil questionnaire. At the beginning, the participants were told that the aim of the study was to adapt for Russian-speaking population a questionnaire originally composed in English. It was highlighted that there were no right or wrong answers for the items. The participants were assured that all collected data would be confidential and anonymous and that no individual data would be analyzed.

#### Statistical analysis and predictions

At this stage, the primary goal was to obtain the clearest and the most interpretable factor structure, therefore we used principal component analysis (PCA) as a factor extraction technique with varimax rotation [57]. All statistical analyses were performed using Statistica 10.0 software. After assessing the PCA component structure each individual item was to meet each of three preset criteria in order to be included into a brief operational Russian TEAQ version: 1) an item exclusion should lead to decrease of overall Cronbach's α; 2) an item should have the highest loading of at least 0.4 for any component [58]; 3) the two highest loadings of an item should not be too similar (a difference of at least 0.1 was required).

We expected that as a result of Study 2 we would compose a reasonably brief questionnaire of 30 to 60 items with an easily interpretable factor structure reflected in 3 to 7 subscales; the factor structure was expected to be reasonably similar to the factor structure of the original English version of the TEAQ, with one or more PCA components corresponding to each of the major domains of childhood touch, current touch, and attitudes to different touch-related behaviors.

### 2.2. Results and discussion

Cronbach’s alpha for the complete 85 item set was high (0.935) demonstrating high level of items’ consistency, with an average inter-item correlation of 0.157. The Kaiser-Meyer-Olkin Measure of Sampling Adequacy value was 0.901 with significance level for Bartlett’s Test of Sphericity ≤ 0.001, therefore the dataset was considered fit for PCA.

#### Principal component analysis

According to Cattell’s scree test [56], five component decision was selected for detailed analysis. Eigenvalues for this solution are presented in Table 1. We can see that five components account for 41.8% of the variance with the largest eigenvalue for the first component (18.93). The latter components have very similar eigenvalues of 5.32 to 3.14.

**Table 1:** Eigenvalues and percentage of variance explained for the 5-factor solution (Stage 2).

	Eigenvalue	Total variance (%)	Cumulative eigenvalue	Cumulative total variance (%)
Factor 1	18.93	22.27	18.93	22.27
Factor 2	5.32	6.26	24.25	28.53
Factor 3	4.48	5.27	28.74	33.81
Factor 4	3.62	4.26	32.36	38.07
Factor 5	3.14	3.70	35.51	41.78

According to the content of items loading highest on each factor, the five component solution yielded an easily interpretable factor structure. Consistent to the predictions, there were separate components for childhood touch experiences (ChT subscale, e.g. “My parents regularly cuddled me as a child”; “As a child I would often hug family members”) and for current touch. Only items related to intimate touch scored high on this component therefore the subscale was defined as Current Intimate Touch (CIT subscale, e.g. “Most days I get a hug or a kiss”, “I can always find somebody to physically comfort me when I am upset”). Three components reflected attitudes to different kinds of affective touch events: attitude to intimate touch (AIT subscale, e.g. “I find a hug very comforting when I am upset”; “I like to stroke the skin of someone I know intimately”), general attitude to friendly social touch and to touch with friends and relatives (Attitude to Friendly Touch or AFT subscale, e.g. “I enjoy having my skin groomed by other people”, “Physical contact with other people is important to me”), and attitude to self-care (ASC subscale, e.g. “I like using body lotions”, “I like the feel of shower gels against my skin”).

Analysis of individual item loadings and effects of their exclusion on Cronbach's α reveals that only 37 items matched all the three inclusion criteria. The 37-item version had very high consistency (Cronbach’s α = 0.9201) with average inter-item correlation of 0.24. Each subscale also had high consistency (all Cronbach's α above 0.82). The paper and pencil version of the TEAQ-37 Rus with scoring instructions is also provided in Supporting information (S3 Table). Copyright of the TEAQ-37 Rus remains with the authors.

For all the items of the TEAQ-37 Rus the factor loadings, item-total correlations, and Cronbach's α if deleted are provided in Supporting information (S4 Table) for the Study 2 sample.

There were several groups of items that failed to integrate into this factor structure during Studies 1 and 2, one of such groups including attitudes to touch interactions with unfamiliar or less familiar people. Very few comparative studies of nonverbal behavior assessing Russians have been published in international peer reviewed journals, but the existing data point that according to Hall’s classification modern Russian culture is predominantly non-contact [59, 60], with particular reservation towards physical contacts with strangers; unfamiliar touch that occurred quite frequently during Soviet times in crowded places and public traffic can be unwillingly tolerated but never sought [61]. Another possible culture-specific facet of item selection may be related to items related to hugs occurring in different contexts: most items concerning habitual use of hugs as an informal greeting were excluded (i.e. “I always greet my friends and family by giving them a hug” or “I usually hug my family and friends when I am saying goodbye”) but the majority of items concerning hugs as emotionally meaningful interactions were retained and included into either AIT subscale (“Hugging someone is a good way of consoling them”, “Sometimes I just need to be hugged”) or into CIT subscale (“Most days I get a hug or a kiss”). In Russian culture hugs are reserved for closer friends and are often used in a more intimate manner, not as a social greeting but as a genuine gesture of affection or consolation [60]. Opposite is true for handshakes that are a very common formal or semi-formal greeting, but normally used between men only (possibly by women but usually on very formal occasions); this is reflected in the results of a post-hoc ANOVA for a handshake related item (“I often shake hands with people”) showing a very robust effect of gender (F = 119.40, *p*<0.001) with mean value for the item for females of 2.52 (SD = 1.27, Mode = 1), and for males of 3.86 (SD = 1.26, Mode = 5). Overall, such gender differences raised a concern that the unequal male to female ratio in our sample would possibly compromise the item composition and the factor structure. Separate exploratory factor analyses were run for males and females, and the differences were found to be very minor, reflecting no significant influence on the item composition and the factor structure of the TEAQ-37 Rus due to the sample gender composition.

In summary, Study 2 led to the construction of a 37-item Russian version of the TEAQ (TEAQ-37 Rus) which was characterized by high internal consistency and a clear five-factor structure (Attitude to Friendly Touch (AFT), Childhood Touch (ChT), Attitude to Self-Care (ASC), Current Intimate Touch (CIT), and Attitude to Intimate Touch (AIT)). The TEAQ-37 Rus was suggested as an operational version for Studies 3 and 4 (confirmatory factor analysis and validating the TEAQ-37 Rus against other psychometric measures). Psychometric properties of the TEAQ-37 Rus will be reported in details according to the data obtained from the confirmation sample (Study 3), to eliminate possible interference of the responses to the items of the TEAQ-37 Rus with responses to the items excluded from further analyses during Study 2.

## Study 3

At this stage of the research we aimed to confirm internal consistency and the validity of the previously obtained factor structure of the 37-item version of Russian TEAQ (TEAQ-37 Rus) with confirmatory factor analysis (CFA) using the data collected from the third sample of Russian speaking participants and to describe general psychometric properties of this version of the questionnaire.

### 3.1 Methods

#### Participants

**Fig 1:**
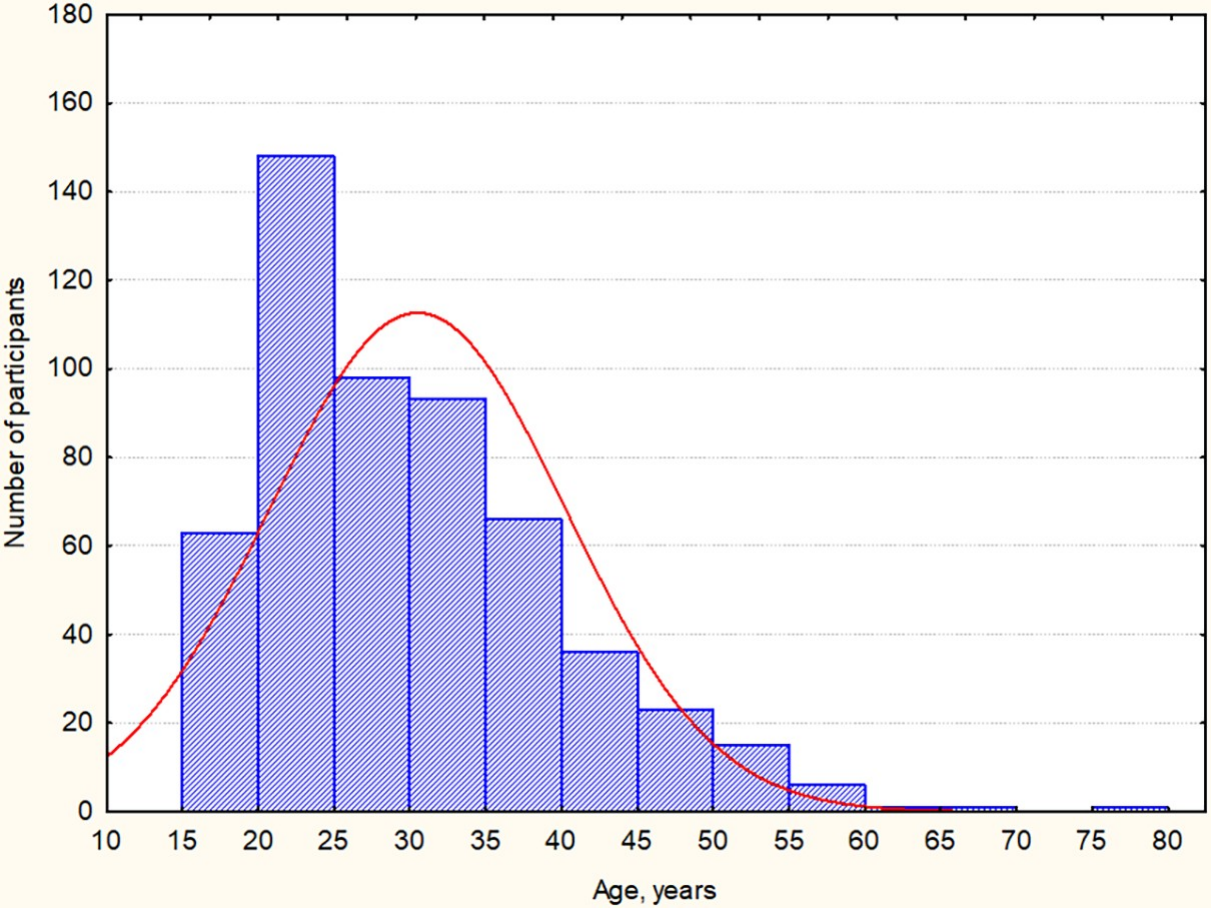
Age distribution for Study 3 sample.

To increase the ecological validity of the CFA sample the data collection was performed by two methods: a) Group A: a highly controlled version of snowball sampling as described above, providing minimal participation bias, 280 participants (167 female, 113 male); and b) Group B: data collected through an internet survey to increase the coverage of different social and age groups, 271 participant (209 female, 62 male). For the purposes of Study 3 both samples were included in a general sample and analyzed together. The total sample included 551 participants (376 female, 68%), with no missing TEAQ-37 Rus, age, or gender data for any of the participants. Participants age varied between 16 and 79 years (M = 30.5, SD = 9.76), age distribution across the sample is reported in Fig 1. Male and female samples did not differ significantly in terms of age (*p* = 0.54); mean age and SDs were also similar (Female: Mean = 30.69, SD = 10.21; Male: Mean = 30.14, SD = 8.71).

#### Materials and procedure

All the data for the Study 3 were collected through online forms. The participants from Group A completed the forms at a room at the university, the participants from Group A completed the forms at home. The participants completed the TEAQ-37 Rus along with several other psychometric tools to assess construct and concurrent validity of the TEAQ-37 Rus within Study 4, so the samples for studies 3 and 4 were the same. For the details on other questionnaires and psychometric instruments used see Study 4, Methods. The composition of the questionnaires for different subsamples was different in order to keep the assessment time within reasonable limits. Total average assessment time did not exceed 30–35 minutes for any subsample. The participants within each subgroup were randomly assigned to one of 4 questionnaire sequences with counterbalanced order of questionnaires. According to the collected feedback, all the questionnaires and the whole procedure was tolerated well.

#### Statistical analysis

CFA was performed in AMOS 21.0.0 software using method of maximal likelihood. The criteria used to determine goodness of model fit were a Root Mean Square Error of Approximation (RMSEA), a Comparative Fit Index (CFI), Relative chi-square (CMIN/DF), and Non-normed fit index NNFI (TLI) [62].

Re-assessment of the factor structure was also performed at this stage to report Cronbach's α and factor loadings for all the items for the TEAQ-37 Rus for the validation sample. Factor analysis settings were identical to Study 2 (PCA as factor extraction technique, Varimax rotation. Distribution assessments (Kolmogorov-Smirnov test) and subscale cross-correlation analysis were performed to evaluate general psychometric properties of the subscales.

### 3.2. Results and discussion

**Fig 2:**
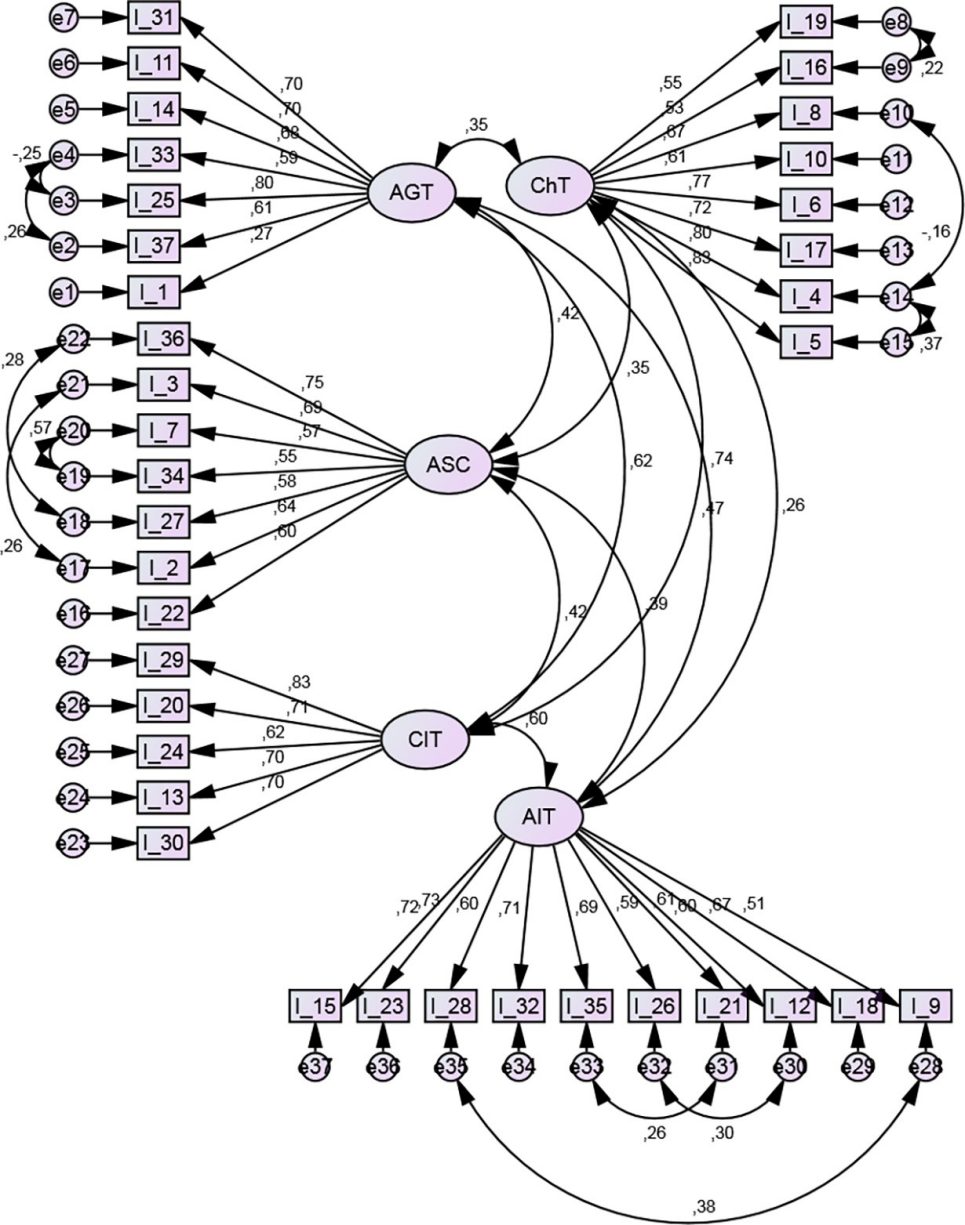
CFA Path diagram for Model 2 of the TEAQ-37 Rus. Rectangles indicate measured variables and large ellipses represent TEAQ-37 Rus subscales. Covariances of errors between items with similar content are shown.

***CFA*.** Initial analysis was performed for a five-factor model where each item loaded for only one factor, with no consideration for possible loadings for two factors and variances of errors for individual items. This model demonstrated nearly satisfactory fit (see Model 1 in Table 2). A modified Model 2 considering covariances of errors for items with similar content (item pairs 33–25, 33–37, 36–27, 3–2, 7–34, 28–9, 35–21, 26–12, 19–16, 8–4, 4–5) demonstrated satisfactory fit (Table 2) [62]. The path diagram for the CFA is provided at Fig 2.

**Table 2:** CFA fit indices of assessed models (Stage 3). CMIN/DF—Relative chi-square; CFI -comparative fix index; NNFI (TLI)—non-normed fit index; RMSEA—root mean square error of approximation.

Model	CMIN/DF	CFI	NNFI (TLI)	RMSEA
1	3.809	.817	.803	.071
2	2.922	.877	.865	.059

#### Replication of the original factor structure and reporting general psychometric properties

The principal component analysis repeated for Study 3 sample corresponded very closely to the results of the CFA; the same five components were observed as for Study 2 sample: Attitude to Friendly Touch (AFT), Childhood Touch (ChT), Attitude to Self-Care (ASC), Current Intimate Touch (CIT), and Attitude to Intimate Touch (AIT). The 5-factor model explained 54% of the total variance. The item loads were very good to moderate, the worst load being 0.427 and the next worst being 0.499. General scale reliability and factor reliabilities were high (total Cronbach's α = 0.920, Cronbach's α for the factors ranging from 0.88 to 0.83). The factor loads for all the items, Cronbach's α, and percentage variance explained for all the subscales are shown in Table 3.

**Table 3:** TEAQ-37 Rus factor structure. Factor loading of each item are shown. (R) after item numbers denotes reverse scored items. At the bottom of the table Cronbach's α and percentage variance explained by each factor are given.

Items of the TEAQ-37 Rus, with numbers		AFT	ChT	ASC	CIT	AIT
31. I enjoy having my skin groomed by other people		***0*.*72***	0.04	0.08	0.25	0.16
11. Physical contact with other people is important to me.		***0*.*66***	0.12	0.15	0.12	0.28
14. I enjoy grooming other people’s skin.		***0*.*66***	0.07	0.06	0.18	0.23
33. I am on huggable terms with quite a few people		***0*.*63***	0.14	0.11	0.09	0.18
25. In general, I would describe myself as a physically affectionate person.		***0*.*51***	0.12	0.12	0.36	0.42
37. I like it when my friends and family greet me by giving me a hug.		***0*.*50***	0.21	0.27	0.04	0.42
1 (R). I dislike people being very physically affectionate towards me.		***0*.*43***	-0.01	-0.09	0.06	0.04
5. My parents regularly cuddled me as a child		0.08	***0*.*85***	0.02	0.14	-0.00
4. There was a lot of physical affection during my childhood		0.06	***0*.*79***	0.10	0.20	-0.04
17. As a child my parents always comforted me when I was upset		-0.04	***0*.*78***	0.02	0.11	0.02
6. As a child I would often hug family members		0.22	***0*.*73***	0.08	0.13	0.09
10. As a child my parents would tuck me up in bed every night and give me a hug and a kiss goodnight		0.03	***0*.*69***	0.07	-0.01	0.09
8. As a child I found a hug from my parents when I was upset made me feel much happier		0.17	***0*.*64***	0.18	0.09	0.19
16. My mother regularly bathed me as a child		-0.05	***0*.*61***	0.22	0.03	0.11
19. As a child my parents would often hold my hand when I was walking along with them.		0.04	***0*.*60***	0.13	0.12	0.17
36. I like to use face masks on my skin		0.13	0.11	***0*.*78***	0.12	0.06
3. I like using body lotions		0.14	0.14	***0*.*70***	0.11	0.01
7. I like to use bath essence when having a bath		0.00	0.11	***0*.*68***	0.05	0.18
34. I like having a bath with lots of bubble bath.		-0.01	0.06	***0*.*67***	0.10	0.15
27. I like exfoliating my skin		0.13	0.05	***0*.*66***	0.14	-0.02
2. I like using moisturisers on my skin		0.08	0.14	***0*.*65***	0.07	0.13
22. I like the feel of shower gels against my skin.		0.09	0.06	***0*.*63***	0.07	0.17
29. I often have my skin stroked.		0.26	0.22	0.11	***0*.*78***	0.09
20. Most days I get a hug or a kiss.		0.15	0.16	0.15	***0*.*70***	0.12
24. I often share a romantic kiss		-0.07	0.12	0.10	***0*.*67***	0.38
13. I can always find somebody to physically comfort me when I am upset		0.13	0.29	0.10	***0*.*64***	0.16
30. I often hold hands with someone I am fond of.		0.35	0.12	0.15	***0*.*57***	0.28
15. I enjoy being cuddled by someone I am fond of		0.24	0.09	0.01	0.04	***0*.*73***
23. I enjoy holding hands with someone I am fond of		0.13	-0.02	0.17	0.26	***0*.*69***
28. Kissing is an enjoyable part of expressing romantic feeling		-0.08	0.07	0.04	0.35	***0*.*67***
32. I like to stroke the skin of someone I know intimately		0.14	0.01	0.05	0.36	***0*.*64***
35. I find a hug very comforting when I am upset		0.34	0.10	0.25	0.08	***0*.*63***
26. It’s good to console people you know well with strokes and hugs		0.21	0.08	0.03	0.07	***0*.*63***
21. Sometimes I just need to be hugged		0.27	-0.02	0.20	-0.04	***0*.*63***
12. Hugging someone is a good way of consoling them.		0.36	0.12	0.10	-0.01	***0*.*59***
18. I enjoy the feeling of my skin against someone else's if I know them intimately		0.22	0.04	0.03	0.27	***0*.*58***
9. Kissing is a great way of expressing physical attraction.		-0.02	0.15	0.04	0.26	***0*.*56***
***Total variance explained***	***0*.*54***	0.09	0.12	0.10	0.09	0.14
***Cronbach's α***	***0*.*92***	0.84	0.85	0.84	0.83	0.88

The confirmatory analyses yielded results proving adequate face validity and internal consistency of the 37-item version of the questionnaire (TEAQ-37 Rus). This version is therefore treated as an operational Russian version of the TEAQ in this manuscript and all the further statistical analyses in Study 3 and Study 4 are performed for the TEAQ-37 Rus. All the resulting subscales are scored and named according to the initial factor analysis and CFA results: Attitude to Friendly Touch (AFT), Childhood Touch (ChT), Attitude to Self-Care (ASC), Current Intimate Touch (CIT), Attitude to Intimate Touch (AIT). The total TEAQ-37 Rus score is calculated as the sum of the subscale scores.

Mean TEAQ-37 Rus score for the sample was 122.33 (SD = 22.15), there were no participants who got highest or lowest possible score (185 or 37), so no floor or ceiling effect was observed. The total TEAQ-37 Rus score distribution for the Study 3 data sample was assessed as not differing significantly from normality (K-S test, p>0.1). The distributions of all the subscales was also normal or close to normal (p>0.001 for all the subscales). No prominent ceiling or floor effects was observed for any subscale. The most prominent skewness and the largest ceiling effects (7.63%) were observed for AIT subscale, indicating that gentle touch between close people is generally perceived as very pleasant by the majority of our participants.

All the subscales significantly correlated with each other (all p < 0.0001), with low to moderate strength of the observed correlations (see Table 4). Attitude to personal grooming correlated least with other components and current social touch correlated most. The strongest correlation was between AFT and AIT (r = 0.62). The weakest correlation was between ChT and AIT (r = 0.25).

**Table 4:** TEAQ-37 Rus subscale data. Mean and standard deviations are provided for subscale score sums, and correlation coefficient values are given for correlations between the subscales.

	Means	SD	AFT	ChT	ASC	CIT	AIT
**AFT**	22.63	5.765	-	0.30	0.33	0.49	0.62
**ChT**	25.05	7.423	0.30	-	0.30	0.40	0.25
**ASC**	18.68	5.671	0.33	0.30	-	0.33	0.33
**CIT**	15.80	5.164	0.49	0.40	0.33	-	0.53
**AIT**	40.35	7.099	0.62	0.25	0.33	0.53	-

## Study 4

At this stage of the research we aimed to test experimental hypotheses 2, 3, and 4, by identifying possible demographic differences in TEAQ-37 Rus responses and by assessing construct and criterion validity of the TEAQ-37 Rus. For general details of the sample composition and the experimental procedure see Study 3, Methods.

At the beginning of Study 4, after obtaining and validating the factor structure of the TEAQ-37 Rus, and after assessing the data on the English version of the TEAQ [51], it was possible to formulate and to put to test more specific experimental hypotheses to further expand previously formulated general experimental hypotheses 2 and 3 (see Introduction):

2.1) Female participants would have higher general TEAQ-37 Rus score, and particularly higher score at ASC TEAQ-37 Rus subscale;
2.2) The correlation between age and attitudes toward social touch would be insignificant or relatively small, though for experience-related subscales there may be a difference between different age groups, particularly for childhood experience, due to gradually improving attitude to nurturing family touch from 1970-1980s to 1990-2000s [4, 61]; education would have little to no effect on TEAQ-37 Rus score;
2.3) People living alone would score lowest on current intimate touch, and people living with partners would score highest;
3.1) In terms of convergent and discriminant validity measured against the Big Five factors, the TEAQ-37 Rus subscales would have insignificant to low strength correlations with the Big Five factors, except for Extraversion and Openness factors that would have low to moderate strength positive correlations with the TEAQ-37 Rus subscales;
3.2) There would be weak to moderate positive correlation with emotional intelligence for the TEAQ-37 Rus subscales.

### 4.1. Participants and methods

#### Demographics

Age and gender effects were assessed for all of the Study 4 sample participants (n = 551). For the majority of the participants data were collected for education (n = 399); most participants had higher education (n = 276), 77 participants had unfinished higher education, and 46 participants had general school or vocational school education. Cohabiting status was assessed for 325 participants (243 female, 82 male), response options were “Living alone” (n = 56), “With a spouse/partner” (n = 151), and “With relatives other than a spouse/partner, or with friends/peers” (n = 147).

#### Psychometric measures

The TEAQ-37 Rus and demographic assessment questions preceded several other psychometric tools to assess construct and concurrent validity of the TEAQ-37 Rus. Different combinations of psychometric instruments were used for different population subsamples to provide a range of measures to validate against, keeping in mind that total assessment time should not exceed 30–35 minutes for any participant. To the best of our knowledge, the TEAQ-37 Rus is the only psychometric measure in Russian that assesses attitudes to and experiences of social touch, with reported factor structure and psychometric properties, therefore it was not possible to validate it against established touch-related self-report questionnaires. To assess the discriminant validity of the TEAQ-37 Rus, we have collected data on personality traits according to the Big Five model, and on EmIn measure of emotional intelligence. To assess the criterion validity of the TEAQ-37 Rus and to further assert the link between the psychometric measures of touch and the C-tactile system, the TEAQ-37 Rus was also validated against the Affective Touch Video clips. A sample of 325 participants (243 female, 82 male) completed the TEAQ-37 Rus, NEO-FFI, and viewed Affective Touch Video clips (always in this particular sequence); a smaller sample of 74 participants completed the TEAQ-37 Rus and EmIn.

#### Big five personality trait assessment

Big Five personality model [63] was used for cross-validation as one of the most widely used personality models focusing on personality traits related to social performance. There are several questionnaires in Russian assessing the Big Five personality traits developed for adults [64]. The most popular and better validated versions are adaptations of the NEO-PI-R and the NEO-FFI [65], an adaptation of Goldberg’s 100-item IPIP scale [66], and yet another Russian version of the NEO-FFI [67, 68]. The latter Russian version of NEO-FFI was selected for the purposes of the study as it is reasonably brief and its factor structure has been extensively replicated on different samples [69, 70].

#### EmIn questionnaire

EmIn questionnaire was selected to measure emotional intelligence as it the most widely used and thoroughly validated Russian questionnaire for self-assessment of emotional intelligence [71–75]. It is composed of 46 items and provides general score for self-assessed emotional intelligence, and subscale scores for Emotion Recognition (ability to recognize emotions in self and others), Emotion Management (ability to manage the emotional state of self and others), Interpersonal Emotional Intelligence (ability to recognize and manage emotions of others), and Self-directed Emotional Intelligence (ability to recognize and manage own emotions).

#### Affective touch video clips

To test the experimental hypothesis 4 and to assess criterion validity of the TEAQ-37 Rus, for a population subsample we administered short video clips depicting actors being touched by another person at different velocities and at different body sites. Subjective ratings of perceived pleasantness of the touch (325 participants; 243 female, 82 male) were recorded. The video set used for the present study were similar to the set developed earlier by Walker and colleagues (2017) but was significantly expanded: there were two actor pairs (a male touching a female and a female touching a male), three velocities (static touch, slow strokes with a velocity of 5 cm/s, and fast strokes with a velocity of 30 cm/s), and eight different body skin sites being touched (palm, hand, dorsal and ventral forearm, upper arm, back, side of the face, and back of the head), 48 videos total. All the videos were 6 s long, had original quality of Full HD (1920×1080 pixels) at 25 fps rate, and were presented at 240 p YouTube quality. Close up angles were used in order not to reveal the faces of the actors, to make the videos less personal. Examples of the videos in YouTube quality are provided in Supporting information (S1–S3 Videos), and the whole video set is available on request. The videos were presented in four randomly assigned counterbalanced sequences. After watching each video clip the participants rated the perceived pleasantness of the touch for the person being touched, on a Likert scale from 1 (very unpleasant) to 7 (extremely pleasant). It has been previously demonstrated that videos depicting slow strokes are consistently rated as the most pleasant kind of touch for hairy skin sites (Walker et al., 2017).

#### Statistical analysis

According to the results of distribution tests (see Study 3) and taking into account large sample sizes, the distributions were close enough to normality to justify the use of parametric statistics for correlations and between-group comparisons for total TEAQ-37 Rus scores and subscale scores, therefore Pearson’s correlation coefficients (*r*) were used. Bonferroni correction was applied as appropriate for all multiple comparisons where specific predictions had not been formulated.

One way between group ANOVAs were used to assess the effects of gender (Gender Group (2)), cohabiting status (Cohab Group (3)) and education (Education Group (3)) on the TEAQ-37 Rus subscale scores for each subscale. To evaluate the relationship between TEAQ-37 Rus scores and perceived pleasantness of touch in touch video clips we had divided the sample into two groups based on TEAQ-37 Rus total scores, median split: TEAQ-37 Rus < 122 (n = 167) and TEAQ-37 Rus ≥ 122 (n = 170). Omnibus repeated measures ANOVA (TEAQ Group (2) * Velocity (3) * Site (8) * Actor Pair (2)) was used to evaluate relations between TEAQ-37 Rus score and perceived pleasantness of touch depicted in video clips. Greenhouse-Geisser sphericity corrections were used where appropriate (corrected *p* values are provided). Scheffé’s post hoc tests were used as both within-group and between-group comparisons were of interest.

### 4.2. Results

#### Demographic group effects

Means and SDs for all the TEAQ-37 Rus subscales for gender, education and cohabiting status groups are provided in Table 5.

**Table 5:** Demographic group data for Study 4.

	Gender				Cohabiting Status						Education					
	Female (N = 376)		Male (N = 175)		Single (N = 58)		With Relatives/Friends (N = 128)		With a Partner (N = 151)		School (N = 46)		Unfinished Higher (N = 77)		Higher (N = 276)	
Subscale	Mean	SD	Mean	SD	Mean	SD	Mean	SD	Mean	SD	Mean	SD	Mean	SD	Mean	SD
**AFT**	23.13	5.84	21.58	5.46	21.47	7.10	22.59	6.17	22.77	6.09	21.96	6.63	21.62	6.17	23.17	6.17
**ChT**	25.45	7.85	24.18	6.36	23.60	6.49	23.41	7.70	23.97	7.74	23.92	8.28	25.22	8.20	24.22	7.30
**ASC**	20.26	5.15	15.28	5.24	16.95	4.63	18.62	5.57	17.81	5.81	18.35	5.73	18.25	5.30	18.23	5.86
**CIT**	16.38	5.11	14.57	5.08	12.14	4.83	13.70	5.31	17.64	4.53	14.53	6.07	15.32	5.54	15.92	5.25
**AIT**	41.15	6.89	38.63	7.25	39.53	6.91	40.42	8.17	41.56	6.20	40.86	7.63	40.03	7.98	41.22	6.86

**Gender.** According to the ANOVAs for the TEAQ-37 Rus subscales, female participants scored significantly more for Attitude to Self-Care (*p*_corr_ < 0.001), Attitude to Friendly Touch (*p*_corr_ = 0.016), Attitude to Intimate Touch (*p*_corr_ < 0.001), and Current Intimate Touch (*p*_corr_< 0.001) subscales; there were no relations between Gender and Childhood touch (*p*_corr_ = 0.21). The most robust Gender effect, consistent with the predictions, was observed for Attitude to Self-Care (see Table 5).

**Age.** A correlation of low strength but of relatively high significance due to large sample size (r = -0.16, *p*_corr_ = 0.001) was observed for Childhood Touch subscale reflecting that participants of older cohorts tended to receive slightly less affective touch in their childhood. No significant correlations with Age were observed for any other TEAQ-37 Rus subscale (all *rs* < 0.06, all *p*s_uncorr_ > 0.15).

**Cohabiting status.** Between group ANOVAs revealed that the effect of Cohabiting status was significant only for Current Intimate Touch subscale (F (2, 322) = 35.19, *p*_corr_ = 0.001, η_p_^2^ = 0.18), pointing that, as expected, participants living with spouses or partners had the highest amount of tactile interactions with close people, and participants living alone had the lowest CIT score (see Table 5). No significant effects were observed for any other TEAQ-37 Rus subscale (all *p*s_uncorr_ > 0.10).

**Education.** Between group ANOVAs revealed no significant effect of education level on any TEAQ-37 Rus subscale (all *p*s_uncorr_ > 0.1).

#### Validation of TEAQ-37 Rus against other psychometric measures

**Big Five personality factors.** The correlations of the TEAQ-37 Rus subscales with the Big Five personality factors are given in Table 6. Consistent with the predictions, the strongest correlations were observed for Extraversion (*r* values ranging from 0.47 for AFT subscale to 0.20 for ASC subscale). Weak but significant correlations with all the TEAQ-37 Rus subscales were observed for Openness (*r* ranging from 0.25 to 0.18). For Agreeableness weak significant correlations were observed for AFT, ChT, CIT, and AIT (*r* ranging from 0.30 to 0.18) but not for ASC. Conscientiousness correlated with CIT only, and Neuroticism correlated with AFT only. All the significant correlations with the Big Five personality factors were positive for all the TEAQ-37 Rus subscales.

**Table 6:** Correlations of the TEAQ-37 Rus subscales with the Big Five personality factors (*r* values).

	Neuroticism	Extraversion	Openness	Agreeableness	Conscientiousness
**AFT**	0.01	**0.47**	**0.25**	**0.30**	-0.02
**ChT**	-0.07	**0.27**	**0.17**	**0.18**	0.00
**ASC**	**0.21**	**0.20**	**0.20**	0.09	0.05
**CIT**	-0.05	**0.44**	**0.22**	**0.18**	**0.15**
**AIT**	0.06	**0.38**	**0.23**	**0.23**	0.03
TEAQ-37 Rus Total	0.04	**0.49**	**0.30**	**0.28**	0.05

**Emotional intelligence (EmIn).** The correlations of the TEAQ-37 Rus subscales with different facets of emotional intelligence, as measured by EmIn, are given in Table 7. All the significant correlations with the EmIn subscales were positive for all the TEAQ-37 Rus subscales. Consistent with the predictions, there was a significant correlation of moderate strength (*r* = 0.33) between total TEAQ-37 Rus score and total EmIn score. While all the TEAQ-37 Rus subscales had positive significant correlations with Interpersonal Emotional Intelligence (*r* ranging from 0.30 to 0.54) and with Emotion Recognition (*r* ranging from 0.30 to 0.45), no significant correlations were found for Self-directed Emotional Intelligence, and for Emotion Management the only significant correlation was observed with CIT TEAQ-37 Rus subscale.

**Table 7:** Correlations of the TEAQ-37 Rus subscales with the EmIn subscales (*r* values).

	Interpersonal EI	Self-directed EI	Emotion Recognition	Emotion Management	EmIn Total
**AFT**	**0.39**	0.06	**0.34**	0.16	**0.28**
**ChT**	**0.41**	0.04	**0.31**	0.18	**0.28**
**ASC**	**0.30**	-0.11	**0.30**	-0.07	0.12
**CIT**	**0.54**	0.11	**0.45**	**0.26**	**0.40**
**AIT**	**0.31**	0.04	**0.32**	0.05	0.21
**TEAQ-37 Rus Total**	**0.50**	0.03	**0.44**	0.14	**0.33**

**Fig 3:**
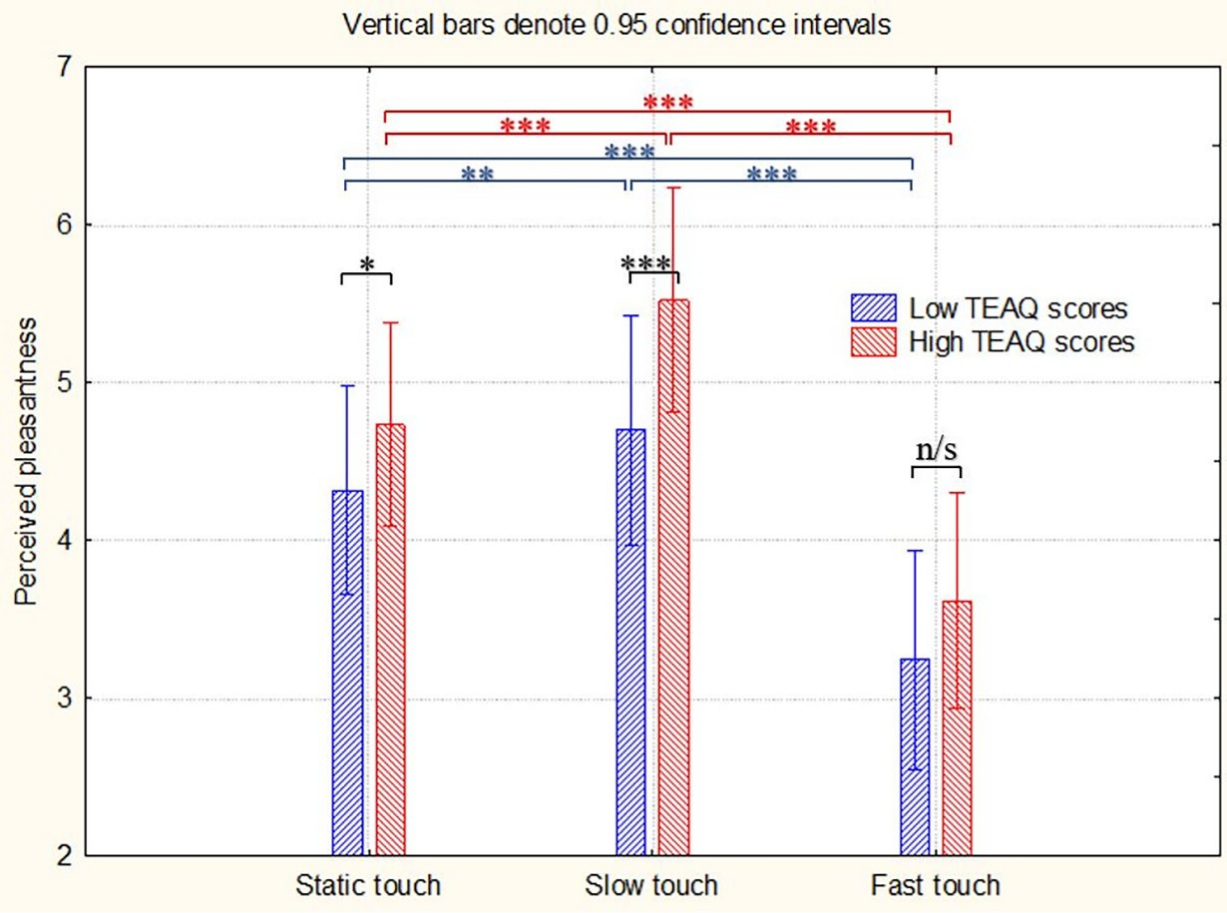
Perceived pleasantness ratings of touch videos for participants with low and high total TEAQ-37 Rus scores. Stars indicate significance levels in post hoc tests (*: *p* < .05, **: *p* < .01, ***: *p* < .001).

**Affective touch video.** In terms of general effects an extremely robust effect of Velocity was observed (F(2, 646) = 419.77, *p*_corr_<0.001, partial eta-squared η^2^ = 0.56) along with highly significant effect of skin site (F(7, 2261) = 55.24, *p*_corr_<0.001, η_p_^2^ = 0.14) and interaction Velocity*Site (F(14, 4522) = 33.50, *p*_corr_<0.001, η_p_^2^ = 0.09) revealing that, according to the expectations, there was a very strong preference of slow strokes compared to fast strokes, and a somewhat smaller but still a very significant preference for slow strokes over static touch for all the sites with hairy skin (for all post hoc comparisons p < .001). In terms of TEAQ-37 Rus group-related effects there was a highly significant effect of Group (F(1, 323) = 27.08, *p*_corr_ <0.001, η^2^ = 0.08) and an interaction Group*Velocity (F(2, 646) = 8.68, *p*_corr_ = 0.001, η^2^ = 0.03). Post-hoc comparisons (Fig 3) indicate that, as predicted, participants with higher TEAQ-37 Rus scores rated all the kinds of touch as more pleasant, with a particularly stronger preference for slow, CT-optimal touch.

## General discussion

### Culture-specific and biologically determined aspects of emotional touch

The aim of this study was to construct a Russian version of the TEAQ questionnaire originally designed in English to assess attitudes to and experience of affective touch and validated on a British population sample [51, 55], and to test the first evidence of its validity and reliability. A large initial pool of 117 touch-related items, after being subject to cultural appropriateness examination and principal component analysis, was reduced to a reasonably compact 37-item questionnaire characterized by good face validity and clear five-factor structure. The factors related to Attitude to Friendly Touch subscale (AFT), Childhood Touch subscale (ChT), Attitude to Self-Care subscale (ASC), Current Intimate Touch subscale (CIT), and Attitude to Intimate Touch subscale (AIT). Very high Cronbach’s α for the whole scale and high Cronbach’s α for all the subscales suggested good reliability. The reliability of the 5-factor structure of the TEAQ-37 Rus was confirmed using CFA with a satisfactory model fit on a separate sample; high values for Cronbach’s α were also replicated. The cohort for this study was characterized by reasonably good age coverage. Due to the nature of the recruitment process the validation sample was somewhat skewed towards university students and people with higher education but there were no noticeable effects of education on TEAQ-37 Rus scores indicating that the TEAQ-37 Rus would yield similar results for people with different social backgrounds within a given culture; further research is needed to provide better estimates for influence of social and subcultural backgrounds on touch-related attitudes and behaviors.

The British version of the TEAQ was constructed and validated on similar samples (618 participants for exploratory factor analysis sample at the item reduction stage, 71.2% female, mean age 26.9; 704 participants for CFA sample, 73.7% female, mean age 27.4), and has a very similar factor structure. There are 57 items yielding 6 factors, with five factors being very closely equivalent to the factors of the TEAQ-37 Rus (childhood touch, friendly touch, attitude to self-care, attitude to intimate touch and current intimate touch). The only factor present in the original English version that has not been reproduced on the Russian samples is ‘Attitude to Unfamiliar Touch’; we would presume that this is probably a consequence of a very reserved attitude towards physical contacts with unfamiliar people and of low incidence of voluntary physical contacts with strangers in Russian culture [61]. If it is indeed the case, tolerance to touch with strangers in Russian-speaking populations may be better accounted not by general attitude to the positive aspects of touch but by other personality traits and attitudes, including attitude to personal boundaries. This explanation is supported by the results from a large cross-cultural study assessing attitudes to acceptability of social touch [31] revealing that Russians use touch in more conservative patterns compared to all the other countries participating in the study (UK, Italy, France and Finland). The factor structure of the TEAQ versions has also confirmed the importance of emotional bond strength revealing that distinct patterns of tactile behavior and attitudes are naturally observed for interactions with emotionally close people, with friends, and with strangers, though preferred and admissible patterns may vary from culture to culture. In general, the data for the British and the Russian samples support our hypothesis 1 that general factor structure of the TEAQ would be similar for different cultures. The nature of the item selection process implemented in the study helps to ensure that each national version is characterized by good content validity for each given language and culture but it may also slightly decrease compatibility of national versions due to larger differences in item content within each subscale. Analysis of this discrepancy supported by further research of touch lexicon (see e.g. [74]) and of possible culture-specific differences of social touch perception and touch-related behaviors would lead to better understanding of culture-related aspects of affective touch. Such understanding would also benefit from research on particularly ‘contact’ (i.e. Southern European or Latin countries) or ‘noncontact’ (some Eastern Asian countries or Native Americans) cultures [53].

Other avenues of research investigating relationships between culture-dependent and biologically determined aspects of emotional touch would be using questionnaire-based measures along with tools providing more direct assessment of physiological and emotional response to touch in settings where the influence of cultural and social context is minimized or manipulated. In the present study we have used a similar approach to assess the construct validity of the TEAQ-37 Rus and to see how TEAQ score is related to perceived pleasantness of person-to person touch depicted in videos with a relatively impersonal and socially neutral context. Participants with higher TEAQ-37 Rus scores rated all kinds of touch as more pleasant, and, according to our initial predictions stemming from a hypothesis of the mediating role of CT-system in affective touch perception [21], had a stronger preference for slow strokes over fast strokes and static touch, fully supporting experimental hypothesis 4. In view of this, the TEAQ-37 Rus seems to be a good screening tool for pre-selecting possible participants with different predisposition towards social touch for further psychophysiological studies of affective touch.

### Social touch, demographic differences, and personality traits

The results of Study 4 fully confirmed our experimental hypothesis 2 and revealed pronounced gender effects and an influence of cohabiting status on current experience of intimate touch. Gender effects should be taken into account when interpreting TEAQ-37 Rus scores, particularly for the Self-Care subscale.

The TEAQ-37 Rus has revealed good discriminative validity when compared against the Big Five personality traits measured with a Russian version of the NEO-FFI. Consistent with our predictions, low to moderate positive correlations were observed between TEAQ-37 Rus subscales, and Extraversion and Openness subscales, thus supporting our experimental hypothesis 3. A somewhat unexpected positive correlation was found between Neuroticism and Attitude to Self-Care (ASC) subscale. A post-hoc explanation can be provided for this correlation, linking higher neuroticism to elevated need for physical acceptance and reassurance which is provided by self-induced activation of the C-tactile system. Indeed, primate behavioral data reveal that inhibition of the endogenous opioid reward system leads to increased need for grooming behavior [75]. Individuals with higher neuroticism and social anxiety may resort to self-grooming as to an easy option: when you feel bad, pamper yourself. Further research on populations with clinical or subclinical levels of anxiety would shed more light on this link.

According to our current understanding of the role of affective touch and CT system in shaping the emotional brain, it was predicted that TEAQ scores would correlate with emotional intelligence. The study confirmed these predictions, yielding robust positive correlations between all the TEAQ-37 Rus subscales (including Childhood Touch), and Emotion Recognition and Interpersonal Emotional Intelligence EmIn subscales (*r* values between 0.30 and 0.54), pointing to a strong link between social touch and empathy. This effect is even more impressive if we take into account that TEAQ-37 Rus contains no items directly related to social competences, and EmIn contains no touch-related items. The number of participants who completed EmIn questionnaire was relatively low though (74 subjects), so these results should be treated as preliminary, and the strength of the link between emotional intelligence and social touch should be confirmed on larger samples.

### Use of the TEAQ-37 Rus for clinical and subclinical populations

The TEAQ-37 Rus was developed with an intent to use it with other psychometric tools and neurobiological measures in order to investigate the role of touch in human emotional well-being, for different clinical and non-clinical populations, including conditions like depression, eating disorders, autism etc. Assessment of the skewness of the subscales revealed that there is no floor-effect for any subscale; it is possible to presume therefore that the TEAQ-37 Rus can be used for clinical and subclinical populations characterized by decreased tolerance for social touch, as with anorexia patients or high functioning autists. Although the TEAQ-37 Rus was initially targeting adult population, inspection of the items’ content reveals no objection to using the TEAQ-37 Rus for teenagers. Further research on more diverse samples is sought but at the moment the TEAQ-37 Rus seems to be a good and flexible enough tool for enhancing our knowledge of importance of nurturing and affiliative touch in both health and disease.

### Other considerations and limitations

The current Russian version of the TEAQ has good overall psychometric properties but some prospects for further refinement can be outlined. The number of questionnaire items for each subscale of the TEAQ-37 Rus is unequal, ranging from 5 to 10 items as a result of following criteria for item retentions that were set prior to discovering the actual factor structure of the TEAQ-Rus. This can be combated by creating a shorter version of the questionnaire as the next step of the research; elimination of the items loading high on several factors and the items with low factor loading may also improve both the factor structure and the model fit. Another aim would be to construct a measure of social touch equally suitable for use in different cultures; this can be achieved at later stages of research after collecting more data for different ‘contact’ and ‘non-contact’ cultures.

### Conclusions

The Touch Experiences and Attitudes Questionnaire is a self-report measure assessing experiences and attitudes in the domain of affective touch. The Russian version constructed in the present study, the TEAQ-37 Rus, has distinct and reliable 5-factor structure, and covers the aspects of general attitude to social touch, attitude to intimate touch, attitude to self-care, current experiences of intimate touch, and memories of touch experiences in childhood. To our best knowledge, the TEAQ is the first available self-report-measure suitable for assessment of affective touch experiences and attitudes for which the factor structure has been determined and validated. We anticipate that this questionnaire will be a valuable tool for researchers of social touch, nonverbal communication, touch perception abnormalities, and the importance of childhood touch experiences for human emotional well-being.

## Supporting information

S1 Table Original 57-item version of the TEAQ with scoring instructions. (DOCX)

S2 Table The complete set of questions in English and in Russian for the initial 117-item TEAQ pool. (DOCX)

S3 Table The TEAQ-37 Rus with scoring instructions (in Russian with English translation provided). (DOCX)

S4 Table TEAQ-37 Rus item data for the Study 2 sample (factor loadings, item-total correlations, and Cronbach's α if deleted). (DOCX)

S1 Video An example of the videos depicting touch interactions (Male to female actor pair, slow strokes, dorsal forearm). (MP4)

S2 Video An example of the videos depicting touch interactions (Female to mail actor pair, fast strokes, hand). (MP4)

S3 Video An example of the videos depicting touch interactions (Female to mail actor pair, static touch, back). (MP4)

S5 Table Stage 1 TEAQ-117 dataset (all the cases). (XLSX)

S6 Table Stage 2 TEAQ-85 dataset (all the cases). Russian text labels are provided for better data traceability.
(XLSX)

S7 Table Stage 3–4 sample (the TEAQ-37 Rus, NEO-FFI, EmIn, Affective Touch Video, and subsample source data, all the cases, Russian text labels are provided for better data traceability). (XLSX)
