# Supplementary material for: Validation and psychometric properties of the Russian version of the Touch Experiences and Attitudes Questionnaire (TEAQ-37 Rus)
Source: PLoS One. 2018 Dec 13;13(12):e0206905. doi: 10.1371/journal.pone.0206905 (PMC6292699; doi:10.1371/journal.pone.0206905)
Supplement: S1 Table — (DOCX) [file pone.0206905.s001.docx]

Touch Experiences and Attitudes Questionnaire (TEAQ)

Please select a response next to each of the statements below to indicate how much you agree or disagree with each statement

|  | Disagree strongly | Disagree a little | Neither agree nor disagree | Agree a little | Agree strongly |
| --- | --- | --- | --- | --- | --- |
| 1. I dislike people being very physically affectionate towards me |  |  |  |  |  |
| 2. I like using body lotions |  |  |  |  |  |
| 3. I have to know someone quite well to enjoy a hug from them |  |  |  |  |  |
| 4. I find it natural to greet my friends and family with a kiss on the cheek |  |  |  |  |  |
| 5. There was a lot of physical affection during my childhood |  |  |  |  |  |
| 6. As a child I would often hug family members |  |  |  |  |  |
| 7. I like to use bath essence when having a bath |  |  |  |  |  |
| 8. I find stroking the hair of a person I am fond of very pleasurable |  |  |  |  |  |
| 9. My parents were not very physically affectionate towards me during my childhood |  |  |  |  |  |
| 10. I like to fall asleep in the arms of someone I am close to |  |  |  |  |  |
| 11. I often snuggle up on the sofa with someone |  |  |  |  |  |
| 12. I enjoy the physical intimacy of sexual foreplay |  |  |  |  |  |
| 13. I like to link arms with my friends and family as I walk along |  |  |  |  |  |
| 14. I usually hug my family and friends when I am saying goodbye |  |  |  |  |  |
| 15. As a child I found a hug from my parents when I was upset made me feel much happier |  |  |  |  |  |
| 16. It’s nice when friends and family members greet me with a kiss |  |  |  |  |  |
| 17. I often hold hands with someone I know intimately |  |  |  |  |  |
| 18. When I am upset, there is usually someone who can comfort me. |  |  |  |  |  |
| 19. Kissing is a great way of expressing physical attraction |  |  |  |  |  |
| 20. It feels really good when someone I am fond of runs their fingers through my hair |  |  |  |  |  |
| 21. I regularly hug people I am close to |  |  |  |  |  |
| 22. As a child my parents would tuck me up in bed every night and give me a hug and a kiss goodnight |  |  |  |  |  |
| 23. My life lacks physical affection |  |  |  |  |  |
| 24. I enjoy having my skin stroked |  |  |  |  |  |
| 25. I often take a shower or bath with someone |  |  |  |  |  |
| 26. I enjoy having sex |  |  |  |  |  |
| 27. I often have sex |  |  |  |  |  |
| 28. I am put off by physical familiarity |  |  |  |  |  |
| 29. I can always find somebody to physically comfort me when I am upset |  |  |  |  |  |
| 30. I always greet my friends and family by giving them a hug |  |  |  |  |  |
| 31. I enjoy being cuddled by someone I am fond of |  |  |  |  |  |
| 32. My mother regularly bathed me as a child |  |  |  |  |  |
| 33. As a child my parents always comforted me when I was upset |  |  |  |  |  |
| 34. I enjoy the feeling of my skin against someone else’s if I know them intimately |  |  |  |  |  |
| 35. As a child my parents would often hold my hand when I was walking along with them |  |  |  |  |  |
| 36. Most days I get a hug or a kiss |  |  |  |  |  |
| 37. If someone I don’t know very well puts a friendly hand on my arm it makes me feel uncomfortable |  |  |  |  |  |
| 38. I often make physical contact with my friends and family when I am with them |  |  |  |  |  |
| 39. It makes me feel uncomfortable if someone I don’t know very well touches me in a friendly manner |  |  |  |  |  |
| 40. I enjoy holding hands with someone I am fond of |  |  |  |  |  |
| 41. I often share a romantic kiss |  |  |  |  |  |
| 42. As a child my mother regularly brushed my hair |  |  |  |  |  |
| 43. I like exfoliating my skin |  |  |  |  |  |
| 44. Kissing is an enjoyable part of expressing romantic feeling |  |  |  |  |  |
| 45. I often have my skin stroked |  |  |  |  |  |
| 46. I often hold hands with someone I am fond of |  |  |  |  |  |
| 47. I like to stroke the skin of someone I know intimately |  |  |  |  |  |
| 48. I am on huggable terms with quite a few people |  |  |  |  |  |
| 49. I often fall asleep while holding someone I am close to |  |  |  |  |  |
| 50. Snuggling up on the sofa with someone is great |  |  |  |  |  |
| 51. I often put my arm around a close friend as we walk along together |  |  |  |  |  |
| 52. I like having a bath with lots of bubble bath |  |  |  |  |  |
| 53. I don’t get many hugs these days |  |  |  |  |  |
| 54. I am often given a shoulder massage |  |  |  |  |  |
| 55. I like to use face masks on my skin |  |  |  |  |  |
| 56. I like it when my friends and family greet me by giving me a hug |  |  |  |  |  |
| 57. I often link arms with my friends and family as I walk along |  |  |  |  |  |

Scoring: Disagree strongly = 1, disagree a little = 2, neither agree nor disagree = 3, agree a little = 4, agree strongly = 5

R denotes items which are reverse scored (i.e. disagree strongly = 5, disagree a little = 4, neither agree nor disagree = 3, agree a little = 2, agree strongly = 1). Item numbers below indicate the items which belong to each of the subscales

Calculate the mean score for each subscale to obtain a subscale score

*Friends and family touch (FFT)* (*11 items*): 4, 13, 14, 16, 21, 30, 38, 48, 51, 56, 57

*Current intimate touch (CIT)* (*14 items*): 11, 17, 18, 23R, 25, 27, 29, 36, 41, 45, 46, 49, 53R, 54

*Childhood touch* (*ChT*) (*9 items*): 5, 6, 9R, 15, 22, 32, 33, 35, 42

*Attitude to self*-*care* (*ASC*) (*5 items*): 2, 7, 43, 52, 55

*Attitude to intimate touch* (*AIT*) (*13 items*): 8, 10, 12, 19, 20, 24, 26, 31, 34, 40, 44, 47, 50

*Attitude to unfamiliar touch* (*AUT*) (*5 items*): 1R, 3R, 28R, 37R, 39R
