# Supplementary material for: Validation and psychometric properties of the Russian version of the Touch Experiences and Attitudes Questionnaire (TEAQ-37 Rus)
Source: PLoS One. 2018 Dec 13;13(12):e0206905. doi: 10.1371/journal.pone.0206905 (PMC6292699; doi:10.1371/journal.pone.0206905)
Supplement: S2 Table — (DOCX) [file pone.0206905.s002.docx]

**Touch Questionnaire**

Please select a number from one to five next to each statement to show how much you agree or disagree with each statement. One represents disagree strongly and five represents agree strongly.

|  |  | **Disagree strongly** | **Disagree a little** | **Neither agree nor disagree** | **Agree a little** | **Agree Strongly** |
| --- | --- | --- | --- | --- | --- | --- |
| 1. | Having an itch scratched is very enjoyable. | 1 | 2 | 3 | 4 | 5 |
| 2. | I dislike people being very physically affectionate towards me. | 1 | 2 | 3 | 4 | 5 |
| 3. | Taking a shower or a bath with someone I am close to is very enjoyable. | 1 | 2 | 3 | 4 | 5 |
| 4. | Sympathetic hugs from people I'm not close to don't console me. | 1 | 2 | 3 | 4 | 5 |
| 5. | I appreciate someone holding my coat for me while I put it on. | 1 | 2 | 3 | 4 | 5 |
| 6. | I like using moisturisers on my skin. | 1 | 2 | 3 | 4 | 5 |
| 7. | Touching some materials really sets my teeth on edge. | 1 | 2 | 3 | 4 | 5 |
| 8. | I like using body lotions. | 1 | 2 | 3 | 4 | 5 |
| 9. | Someone I am fond of often runs their fingers through my hair. | 1 | 2 | 3 | 4 | 5 |
| 10. | I often stroke animals. | 1 | 2 | 3 | 4 | 5 |
| 11. | I have to know someone quite well to enjoy a hug from them. | 1 | 2 | 3 | 4 | 5 |
| 12. | On a bus or train, I feel uncomfortable when a stranger’s arm or leg touches mine. | 1 | 2 | 3 | 4 | 5 |
| 13. | As a child I would frequently play rough and tumble with my friends. | 1 | 2 | 3 | 4 | 5 |
| 14. | I find it natural to greet my friends and family with a kiss on the cheek. | 1 | 2 | 3 | 4 | 5 |
| 15. | People invading my personal space makes me feel uncomfortable. | 1 | 2 | 3 | 4 | 5 |
| 16. | I like to make sexual foreplay last as long as possible. | 1 | 2 | 3 | 4 | 5 |
| 17. | Shoulder massages are very enjoyable. | 1 | 2 | 3 | 4 | 5 |
| 18. | I was tickled a lot as a child. | 1 | 2 | 3 | 4 | 5 |
| 19. | I have oily skin | 1 | 2 | 3 | 4 | 5 |
| 20. | There was a lot of physical affection during my childhood. | 1 | 2 | 3 | 4 | 5 |
| 21. | My parents regularly cuddled me as a child. | 1 | 2 | 3 | 4 | 5 |
| 22. | I’m not a very tactile person. | 1 | 2 | 3 | 4 | 5 |
| 23. | As a child I would often hug family members. | 1 | 2 | 3 | 4 | 5 |
| 24. | I like to use bath essence when having a bath. | 1 | 2 | 3 | 4 | 5 |
|  |  | **Disagree strongly** | **Disagree a little** | **Neither agree nor disagree** | **Agree a little** | **Agree Strongly** |
| 25. | I find stroking the hair of a person I am fond of very pleasurable. | 1 | 2 | 3 | 4 | 5 |
| 26. | My parents were not very physically affectionate towards me during my childhood. | 1 | 2 | 3 | 4 | 5 |
| 27. | I like to fall asleep in the arms of someone I am close to. | 1 | 2 | 3 | 4 | 5 |
| 28. | I often snuggle up on the sofa with someone. | 1 | 2 | 3 | 4 | 5 |
| 29. | While flirting with someone I like to make subtle physical contact with them. | 1 | 2 | 3 | 4 | 5 |
| 30. | I enjoy the physical intimacy of sexual foreplay. | 1 | 2 | 3 | 4 | 5 |
| 31. | I like to link arms with my friends and family as I walk along. | 1 | 2 | 3 | 4 | 5 |
| 32. | I have sex for my pleasure more than my partner's. | 1 | 2 | 3 | 4 | 5 |
| 33. | I usually hug my family and friends when I am saying goodbye. | 1 | 2 | 3 | 4 | 5 |
| 34. | I enjoy getting my hair washed at the hairdressers | 1 | 2 | 3 | 4 | 5 |
| 35. | I often have my skin groomed by a close friend. | 1 | 2 | 3 | 4 | 5 |
| 36. | I often feel lonely. | 1 | 2 | 3 | 4 | 5 |
| 37. | As a child I found a hug from my parents when I was upset made me feel much happier. | 1 | 2 | 3 | 4 | 5 |
| 38. | When having sex I focus on pleasing my partner. | 1 | 2 | 3 | 4 | 5 |
| 39. | It's nice when friends and family members greet me with a kiss. | 1 | 2 | 3 | 4 | 5 |
| 40. | I find taking a shower or bath very enjoyable. | 1 | 2 | 3 | 4 | 5 |
| 41. | I often hold hands with someone I know intimately. | 1 | 2 | 3 | 4 | 5 |
| 42. | When I am upset, there is usually someone who can comfort me. | 1 | 2 | 3 | 4 | 5 |
| 43. | I feel comfortable shaking hands in introduction. | 1 | 2 | 3 | 4 | 5 |
| 44. | Kissing is a great way of expressing physical attraction. | 1 | 2 | 3 | 4 | 5 |
| 45. | It feels really good when someone I am fond of runs their fingers through my hair. | 1 | 2 | 3 | 4 | 5 |
| 46. | I like stroking animals. | 1 | 2 | 3 | 4 | 5 |
| 47. | I cannot wear certain types of clothing because I don’t like the feel of certain materials against my skin. | 1 | 2 | 3 | 4 | 5 |
| 48. | I regularly hug people I am close to. | 1 | 2 | 3 | 4 | 5 |
| 49. | As a child my parents would tuck me up in bed every night and give me a hug and a kiss goodnight. | 1 | 2 | 3 | 4 | 5 |
| 50. | I often shake hands with people. | 1 | 2 | 3 | 4 | 5 |
| 51. | My life lacks physical affection. | 1 | 2 | 3 | 4 | 5 |
| 52. | I enjoy having my skin stroked. | 1 | 2 | 3 | 4 | 5 |
| 53. | I don't like the feel of wool against my skin. | 1 | 2 | 3 | 4 | 5 |
| 54. | Physical contact with other people is important to me. | 1 | 2 | 3 | 4 | 5 |
|  |  | **Disagree strongly** | **Disagree a little** | **Neither agree nor disagree** | **Agree a little** | **Agree Strongly** |
| 55. | I often go to the hairdressers. | 1 | 2 | 3 | 4 | 5 |
| 56. | I often take a shower or bath with someone. | 1 | 2 | 3 | 4 | 5 |
| 57. | I enjoy having sex. | 1 | 2 | 3 | 4 | 5 |
| 58. | I like the feel of velvet against my skin. | 1 | 2 | 3 | 4 | 5 |
| 59. | I enjoy giving people shoulder massages. | 1 | 2 | 3 | 4 | 5 |
| 60. | When I was young, kissing my family in greeting was natural. | 1 | 2 | 3 | 4 | 5 |
| 61. | I have itchy skin. | 1 | 2 | 3 | 4 | 5 |
| 62. | I was alone a lot during my childhood | 1 | 2 | 3 | 4 | 5 |
| 63. | I often have sex. | 1 | 2 | 3 | 4 | 5 |
| 64. | I am put off by physical familiarity. | 1 | 2 | 3 | 4 | 5 |
| 65. | Hugging someone is a good way of consoling them. | 1 | 2 | 3 | 4 | 5 |
| 66. | I can always find somebody to physically comfort me when I am upset. | 1 | 2 | 3 | 4 | 5 |
| 67. | I enjoy grooming other people’s skin. | 1 | 2 | 3 | 4 | 5 |
| 68. | I always greet my friends and family by giving them a hug. | 1 | 2 | 3 | 4 | 5 |
| 69. | I enjoy being cuddled by someone I am fond of. | 1 | 2 | 3 | 4 | 5 |
| 70. | My mother regularly bathed me as a child. | 1 | 2 | 3 | 4 | 5 |
| 71. | I would frequently be given piggy backs as a child | 1 | 2 | 3 | 4 | 5 |
| 72. | I used to get into fights with my classmates at primary school. | 1 | 2 | 3 | 4 | 5 |
| 73. | I like to be in control while having sex. | 1 | 2 | 3 | 4 | 5 |
| 74 | As a child my parents always comforted me when I was upset. | 1 | 2 | 3 | 4 | 5 |
| 75 | I was bullied by my classmates as a child. | 1 | 2 | 3 | 4 | 5 |
| 76 | I enjoy the feeling of my skin against someone else’s if I know them intimately | 1 | 2 | 3 | 4 | 5 |
| 77 | As a child my parents would often hold my hand when I was walking along with them. | 1 | 2 | 3 | 4 | 5 |
| 78 | Most days I get a hug or a kiss. | 1 | 2 | 3 | 4 | 5 |
| 79 | If someone I don’t know very well puts a friendly hand on my arm it makes me feel uncomfortable. | 1 | 2 | 3 | 4 | 5 |
| 80 | I enjoy using massage oils. | 1 | 2 | 3 | 4 | 5 |
| 81 | I often make physical contact with my friends and family when I am with them. | 1 | 2 | 3 | 4 | 5 |
| 82 | I have dry skin. | 1 | 2 | 3 | 4 | 5 |
| 83 | Sometimes I just need to be hugged. | 1 | 2 | 3 | 4 | 5 |
| 84 | I take my time over sexual foreplay. | 1 | 2 | 3 | 4 | 5 |
| 85 | I dislike the feeling of silk against my skin. | 1 | 2 | 3 | 4 | 5 |
| 86 | Quite often I have sex because I feel obliged to. | 1 | 2 | 3 | 4 | 5 |
|  |  | **Disagree strongly** | **Disagree a little** | **Neither agree nor disagree** | **Agree a little** | **Agree Strongly** |
| 87 | I like the feel of shower gels against my skin | 1 | 2 | 3 | 4 | 5 |
| 88 | It makes me feel uncomfortable if someone I don’t know very well touches me in a friendly manner. | 1 | 2 | 3 | 4 | 5 |
| 89 | I like the feeling of fur against my skin. | 1 | 2 | 3 | 4 | 5 |
| 90 | I enjoy holding hands with someone I am fond of. | 1 | 2 | 3 | 4 | 5 |
| 91 | I often share a romantic kiss. | 1 | 2 | 3 | 4 | 5 |
| 92 | In general, I would describe myself as a physically affectionate person. | 1 | 2 | 3 | 4 | 5 |
| 93 | It’s good to console people you know well with strokes and hugs. | 1 | 2 | 3 | 4 | 5 |
| 94 | As a child my mother regularly brushed my hair. | 1 | 2 | 3 | 4 | 5 |
| 95 | I like exfoliating my skin. | 1 | 2 | 3 | 4 | 5 |
| 96 | I like fiddling with my hair. | 1 | 2 | 3 | 4 | 5 |
| 97 | Kissing is an enjoyable part of expressing romantic feeling. | 1 | 2 | 3 | 4 | 5 |
| 98 | I often have my skin stroked. | 1 | 2 | 3 | 4 | 5 |
| 99 | I often hold hands with someone I am fond of. | 1 | 2 | 3 | 4 | 5 |
| 100 | I enjoy having my skin groomed by other people. | 1 | 2 | 3 | 4 | 5 |
| 101 | I like to stroke the skin of someone I know intimately. | 1 | 2 | 3 | 4 | 5 |
| 102 | I am on huggable terms with quite a few people. | 1 | 2 | 3 | 4 | 5 |
| 103 | I often fall asleep while holding someone I am close to. | 1 | 2 | 3 | 4 | 5 |
| 104 | Snuggling up on the sofa with someone is great. | 1 | 2 | 3 | 4 | 5 |
| 105 | I often put my arm around a close friend as we walk along together. | 1 | 2 | 3 | 4 | 5 |
| 106 | I like having a bath with lots of bubble bath. | 1 | 2 | 3 | 4 | 5 |
| 107 | I find a hug very comforting when I am upset. | 1 | 2 | 3 | 4 | 5 |
| 108 | I like squeezing other people's spots. | 1 | 2 | 3 | 4 | 5 |
| 109 | I don't get many hugs these days. | 1 | 2 | 3 | 4 | 5 |
| 110 | I am often given a shoulder massage. | 1 | 2 | 3 | 4 | 5 |
| 111 | I like to use face masks on my skin | 1 | 2 | 3 | 4 | 5 |
| 112 | I like it when my friends and family greet me by giving me a hug. | 1 | 2 | 3 | 4 | 5 |
| 113 | I meet up with my friends and family frequently. | 1 | 2 | 3 | 4 | 5 |
| 114 | I often have an itch scratched. | 1 | 2 | 3 | 4 | 5 |
| 115 | I often link arms with my friends and family as I walk along. | 1 | 2 | 3 | 4 | 5 |
| 116 | I like the feeling of cotton against my skin. | 1 | 2 | 3 | 4 | 5 |
| 117 | I enjoy someone messing with my hair. | 1 | 2 | 3 | 4 | 5 |

**Опросник по изучению тактильного восприятия**

Пожалуйста, оцените каждое утверждение в баллах от одного до пяти, чтобы отразить, в какой степени вы согласны или не согласны с данным пунктом. Один балл означает полное несогласие, а пять баллов – полное согласие с утверждением.

|  | Пункты | **Совершенно не согласен** | **Скорее не согласен** | **Не могу опреде-литься** | **Скорее соглаcен** | **Совершенно согласен** |
| --- | --- | --- | --- | --- | --- | --- |
| 1. | Очень приятно почесать, то, что чешется. | 1 | 2 | 3 | 4 | 5 |
| 2. | Мне не нравиться, когда люди, проявляя свое расположение ко мне, касаются меня. | 1 | 2 | 3 | 4 | 5 |
| 3. | Мне очень нравится принимать душ или ванну вместе с близким мне человеком. | 1 | 2 | 3 | 4 | 5 |
| 4. | Я не испытываю облегчения от сочувствующих объятий людей, которые мне не близки. | 1 | 2 | 3 | 4 | 5 |
| 5. | Мне нравится, когда кто-то придерживает мое пальто, чтобы помочь мне его надеть. | 1 | 2 | 3 | 4 | 5 |
| 6. | Я люблю пользоваться средствами для увлажнения кожи. | 1 | 2 | 3 | 4 | 5 |
| 7. | Прикосновение к некоторым поверхностям и материалам действует мне на нервы. | 1 | 2 | 3 | 4 | 5 |
| 8. | Я люблю пользоваться лосьонами для тела. | 1 | 2 | 3 | 4 | 5 |
| 9. | Человек, который мне нравится, часто перебирает мои волосы. | 1 | 2 | 3 | 4 | 5 |
| 10. | Я часто глажу животных. | 1 | 2 | 3 | 4 | 5 |
| 11. | Мне приятно обниматься только с хорошо знакомыми людьми. | 1 | 2 | 3 | 4 | 5 |
| 12. | В автобусе или в вагоне метро мне не приятно, когда ко мне прикасается рука или нога незнакомого человека. | 1 | 2 | 3 | 4 | 5 |
| 13. | В детстве мне нравилось возиться и бороться с моими друзьями. | 1 | 2 | 3 | 4 | 5 |
| 14. | Мне кажется естественным при встрече поцеловать в щёку своих друзей или родных. | 1 | 2 | 3 | 4 | 5 |
| 15. | Мне не комфортно, когда люди вторгаются в мое личное пространство. | 1 | 2 | 3 | 4 | 5 |
| 16. | В сексе мне нравятся как можно более долгие прелюдии. | 1 | 2 | 3 | 4 | 5 |
| 17. | Массаж плеч – это очень приятно. | 1 | 2 | 3 | 4 | 5 |
| 18. | Меня часто щекотали в детстве | 1 | 2 | 3 | 4 | 5 |
| 19. | У меня жирная кожа. | 1 | 2 | 3 | 4 | 5 |
| 20. | В детстве мне доставалось много физической ласки. | 1 | 2 | 3 | 4 | 5 |
| 21. | Мои родители часто обнимали меня в детстве. | 1 | 2 | 3 | 4 | 5 |
| 22. | Я вообще не очень люблю тактильные контакты. | 1 | 2 | 3 | 4 | 5 |
| 23. | В детстве я часто обнимал родных | 1 | 2 | 3 | 4 | 5 |
| 24. | Я люблю пользоваться пеной или другими средствами для ванны. | 1 | 2 | 3 | 4 | 5 |
| 25. | Мне приятно гладить волосы любимого человека.. | 1 | 2 | 3 | 4 | 5 |
| 26. | Мои родители редко обнимали меня в детстве | 1 | 2 | 3 | 4 | 5 |
| 27. | Мне нравится засыпать в объятьях близкого мне человека. | 1 | 2 | 3 | 4 | 5 |
| 28. | Я часто прижимаюсь к близкому мне человеку, когда мы сидим диване. | 1 | 2 | 3 | 4 | 5 |
| 29. | Когда я флиртую с кем-то, мне нравится легко прикасаться к нему. | 1 | 2 | 3 | 4 | 5 |
| 30. | Мне нравится физическая близость во время прелюдии к сексу. | 1 | 2 | 3 | 4 | 5 |
| 31. | Я люблю держаться за руки во время прогулок со своими друзьями и знакомыми. | 1 | 2 | 3 | 4 | 5 |
| 32. | Я занимаюсь сексом скорее ради собственного удовольствия, чем ради партнера. | 1 | 2 | 3 | 4 | 5 |
| 33. | Прощаясь, я обычно обнимаюсь со своими друзьями и родными. | 1 | 2 | 3 | 4 | 5 |
| 34. | Мне нравится, когда парикмахер моет мои волосы. | 1 | 2 | 3 | 4 | 5 |
| 35. | Я часто позволяю своим близким мыть меня. | 1 | 2 | 3 | 4 | 5 |
| 36. | Я часто испытываю одиночество. | 1 | 2 | 3 | 4 | 5 |
| 37. | В детстве объятия родителей часто помогали мне успокоиться и почувствовать себя лучше. | 1 | 2 | 3 | 4 | 5 |
| 38. | Когда я занимаюсь сексом, я в первую очередь стараюсь доставить удовольствие партнеру. | 1 | 2 | 3 | 4 | 5 |
| 39. | Мне приятно, когда друзья и родные целуют меня при встрече. | 1 | 2 | 3 | 4 | 5 |
| 40. | Мне доставляет большое удовольствие принимать душ или ванну. | 1 | 2 | 3 | 4 | 5 |
| 41. | Я часто держусь за руки со своими близкими. | 1 | 2 | 3 | 4 | 5 |
| 42. | Когда мне плохо, обычно есть человек, который поможет мне успокоиться. | 1 | 2 | 3 | 4 | 5 |
| 43. | Я не испытываю дискомфорта, когда пожимаю руку при знакомстве. | 1 | 2 | 3 | 4 | 5 |
| 44. | Поцелуй – отличный способ выразить, что кто-то тебе привлекателен физически. | 1 | 2 | 3 | 4 | 5 |
| 45. | Мне очень нравится, когда близкий мне человек перебирает мои волосы. | 1 | 2 | 3 | 4 | 5 |
| 46. | Я люблю гладить животных. | 1 | 2 | 3 | 4 | 5 |
| 47. | Я не могу носить одежду из некоторых тканей, потому что мне не нравится, как они ощущаются на коже. | 1 | 2 | 3 | 4 | 5 |
| 48. | Я часто обнимаю своих близких. | 1 | 2 | 3 | 4 | 5 |
| 49. | В моем детстве родители каждую ночь укладывали меня спать, укрывали меня одеялом, обнимали и целовали. | 1 | 2 | 3 | 4 | 5 |
| 50. | Я часто пожимаю руки другим людям | 1 | 2 | 3 | 4 | 5 |
| 51. | Мне в жизни не хватает физической ласки. | 1 | 2 | 3 | 4 | 5 |
| 52. | Мне приятно, когда меня гладят по коже. | 1 | 2 | 3 | 4 | 5 |
| 53. | Мне не нравится ощущать прикосновение шерсти к своей коже. | 1 | 2 | 3 | 4 | 5 |
| 54. | Физический контакт с другими людьми важен для меня. | 1 | 2 | 3 | 4 | 5 |
| 55. | Я часто хожу в парикмахерскую | 1 | 2 | 3 | 4 | 5 |
| 56. | Я часто принимаю душ или ванну с близким мне человеком. | 1 | 2 | 3 | 4 | 5 |
| 57. | Я люблю заниматься сексом. | 1 | 2 | 3 | 4 | 5 |
| 58. | Мне нравится чувствовать прикосновение бархата к коже. | 1 | 2 | 3 | 4 | 5 |
| 59. | Я люблю делать другим людям массаж | 1 | 2 | 3 | 4 | 5 |
| 60. | В детстве для мне было естественным целовать родных при встрече. | 1 | 2 | 3 | 4 | 5 |
| 61. | Моя кожа часто чешется | 1 | 2 | 3 | 4 | 5 |
| 62. | В детстве я часто оставался один (оставалась одна) | 1 | 2 | 3 | 4 | 5 |
| 63. | Я часто занимаюсь сексом. | 1 | 2 | 3 | 4 | 5 |
| 64. | Мне не нравятся прикосновения незнакомых и малознакомых людей | 1 | 2 | 3 | 4 | 5 |
| 65. | Обнять человека – хороший способ утешить, успокоить его. | 1 | 2 | 3 | 4 | 5 |
| 66. | Всегда найдется человек, который обнимет и успокоит меня, когда я расстроен. | 1 | 2 | 3 | 4 | 5 |
| 67. | Мне нравится прикасаться к коже других людей | 1 | 2 | 3 | 4 | 5 |
| 68. | Я всегда обнимаюсь с друзьями при встрече | 1 | 2 | 3 | 4 | 5 |
| 69. | Мне нравится, когда меня обнимает приятный мне человек | 1 | 2 | 3 | 4 | 5 |
| 70. | Моя мама в детстве часто купала меня. | 1 | 2 | 3 | 4 | 5 |
| 71. | В детстве меня часто катали на спине. | 1 | 2 | 3 | 4 | 5 |
| 72. | Я часто дрался с одноклассниками в начальной школе | 1 | 2 | 3 | 4 | 5 |
| 73. | Когда я занимаюсь сексом, мне нравится контролировать процесс. | 1 | 2 | 3 | 4 | 5 |
| 74 | В детстве родители всегда утешали меня, когда я был расстроен | 1 | 2 | 3 | 4 | 5 |
| 75 | В школе мои одноклассники обижали меня | 1 | 2 | 3 | 4 | 5 |
| 76 | Мне нравится чувствовать, как моя кожа касается кожи близкого мне человека | 1 | 2 | 3 | 4 | 5 |
| 77 | В детстве, когда мы гуляли, родители часто брали меня за руку. | 1 | 2 | 3 | 4 | 5 |
| 78 | Почти каждый день я обнимаюсь и целуюсь | 1 | 2 | 3 | 4 | 5 |
| 79 | Мне некомфортно, если кто-то не очень близкий дружески приобнимает меня | 1 | 2 | 3 | 4 | 5 |
| 80 | Я люблю использовать масло для массажа | 1 | 2 | 3 | 4 | 5 |
| 81 | Я часто прикасаюсь к друзьям и родным, когда общаюсь с ними | 1 | 2 | 3 | 4 | 5 |
| 82 | У меня сухая кожа. | 1 | 2 | 3 | 4 | 5 |
| 83 | Иногда мне хочется, чтобы меня обняли. | 1 | 2 | 3 | 4 | 5 |
| 84 | В сексе мне нравится не торопиться, а растягивать прелюдию. | 1 | 2 | 3 | 4 | 5 |
| 85 | Мне не нравится ощущение шелка на коже. | 1 | 2 | 3 | 4 | 5 |
| 86 | Часто я занимаюсь сексом, потому что «надо» | 1 | 2 | 3 | 4 | 5 |
| 87 | Мне нравится ощущение геля для душа на коже. | 1 | 2 | 3 | 4 | 5 |
| 88 | Мне неприятно, когда малознакомый человек пытается по-дружески прикасаться ко мне. | 1 | 2 | 3 | 4 | 5 |
| 89 | Мне нравится ощущать прикосновение меха к своей коже. | 1 | 2 | 3 | 4 | 5 |
| 90 | Мне приятно держаться за руки с человеком, который мне нравится. | 1 | 2 | 3 | 4 | 5 |
| 91 | Я часто целуюсь с любимым человеком | 1 | 2 | 3 | 4 | 5 |
| 92 | В целом я могу описать себя как человека, который любит физические контакты | 1 | 2 | 3 | 4 | 5 |
| 93 | Я считаю правильным обнять и погладить близкого человека, чтобы утешить его. | 1 | 2 | 3 | 4 | 5 |
| 94 | В детстве мама часто расчёсывала мне волосы . | 1 | 2 | 3 | 4 | 5 |
| 95 | Мне нравится делать себе пилинг. | 1 | 2 | 3 | 4 | 5 |
| 96 | Мне нравится играть (накручивать на палец, перебирать пряди)со своими волосами. | 1 | 2 | 3 | 4 | 5 |
| 97 | Поцелуи – приятный способ выразить свои романтические чувства. | 1 | 2 | 3 | 4 | 5 |
| 98 | Меня часто гладят. | 1 | 2 | 3 | 4 | 5 |
| 99 | Я часто держусь за руки с людьми, которые мне нравятся. | 1 | 2 | 3 | 4 | 5 |
| 100 | Мне нравится, когда к моей коже прикасаются другие люди . | 1 | 2 | 3 | 4 | 5 |
| 101 | Мне нравится поглаживать кожу любимого человека. | 1 | 2 | 3 | 4 | 5 |
| 102 | Я обнимаюсь при встрече со многими знакомыми. | 1 | 2 | 3 | 4 | 5 |
| 103 | Я часто засыпаю, обняв любимого человека. | 1 | 2 | 3 | 4 | 5 |
| 104 | Сидеть на диване, обнявшись – здорово | 1 | 2 | 3 | 4 | 5 |
| 105 | Я часто приобнимаю своего близкого друга во время прогулки | 1 | 2 | 3 | 4 | 5 |
| 106 | Мне нравится принимать ванну с большим количеством пены . | 1 | 2 | 3 | 4 | 5 |
| 107 | Меня очень успокаивают объятия, когда я расстроен | 1 | 2 | 3 | 4 | 5 |
| 108 | Я люблю выдавливать прыщи другим людям. | 1 | 2 | 3 | 4 | 5 |
| 109 | В последнее время меня не часто обнимали. | 1 | 2 | 3 | 4 | 5 |
| 110 | Мне часто делают массаж плеч. | 1 | 2 | 3 | 4 | 5 |
| 111 | Мне нравится пользоваться косметическими масками для лица. | 1 | 2 | 3 | 4 | 5 |
| 112 | Мне нравится, когда мои друзья и родные обнимаю меня при встрече | 1 | 2 | 3 | 4 | 5 |
| 113 | Я часто провожу время с друзьями и родными. | 1 | 2 | 3 | 4 | 5 |
| 114 | Я люблю почесываю свою кожу. | 1 | 2 | 3 | 4 | 5 |
| 115 | Я часто держусь за руки с друзьями и родными во время прогулки. | 1 | 2 | 3 | 4 | 5 |
| 116 | Мне нравится ощущать прикосновение хлопка к своей коже. | 1 | 2 | 3 | 4 | 5 |
| 117 | Мне нравится, когда кто-то ерошит мои волосы | 1 | 2 | 3 | 4 | 5 |
