# Supplementary material for: Validation and psychometric properties of the Russian version of the Touch Experiences and Attitudes Questionnaire (TEAQ-37 Rus)
Source: PLoS One. 2018 Dec 13;13(12):e0206905. doi: 10.1371/journal.pone.0206905 (PMC6292699; doi:10.1371/journal.pone.0206905)
Supplement: S3 Table — (DOCX) [file pone.0206905.s003.docx]

**Опросник по изучению тактильного восприятия**

Пожалуйста, оцените каждое утверждение в баллах от одного до пяти, чтобы отразить, в какой степени вы согласны или не согласны с данным пунктом. Один балл означает полное несогласие, а пять баллов – полное согласие с утверждением.

|  |  | **Совершенно не согласен** | **Скорее не согласен** | **Не могу опреде-литься** | **Скорее соглаcен** | **Совершенно согласен** |
| --- | --- | --- | --- | --- | --- | --- |
| 1. | Мне не нравится, когда люди, проявляя свое расположение ко мне, касаются меня. | 1 | 2 | 3 | 4 | 5 |
| 2. | Я люблю пользоваться средствами для увлажнения кожи. | 1 | 2 | 3 | 4 | 5 |
| 3. | Я люблю пользоваться лосьонами для тела. | 1 | 2 | 3 | 4 | 5 |
| 4. | В детстве мне доставалось много физической ласки. | 1 | 2 | 3 | 4 | 5 |
| 5. | Мои родители часто обнимали меня в детстве. | 1 | 2 | 3 | 4 | 5 |
| 6. | В детстве я часто обнимал родных. | 1 | 2 | 3 | 4 | 5 |
| 7. | Я люблю пользоваться пеной или другими средствами для ванны. | 1 | 2 | 3 | 4 | 5 |
| 8. | В детстве объятия родителей часто помогали мне успокоиться и почувствовать себя лучше. | 1 | 2 | 3 | 4 | 5 |
| 9. | Поцелуй – отличный способ выразить, что кто-то тебе привлекателен физически. | 1 | 2 | 3 | 4 | 5 |
| 10. | В моем детстве родители каждую ночь укладывали меня спать, укрывали меня одеялом, обнимали и целовали. | 1 | 2 | 3 | 4 | 5 |
| 11. | Физический контакт с другими людьми важен для меня. | 1 | 2 | 3 | 4 | 5 |
| 12. | Обнять человека – хороший способ утешить, успокоить его. | 1 | 2 | 3 | 4 | 5 |
| 13. | Всегда найдется человек, который обнимет и успокоит меня, когда я расстроен. | 1 | 2 | 3 | 4 | 5 |
| 14. | Мне нравится прикасаться к коже других людей. | 1 | 2 | 3 | 4 | 5 |
| 15. | Мне нравится, когда меня обнимает приятный мне человек. | 1 | 2 | 3 | 4 | 5 |
| 16. | Моя мама в детстве часто купала меня. | 1 | 2 | 3 | 4 | 5 |
| 17. | В детстве родители всегда утешали меня, когда я был расстроен. | 1 | 2 | 3 | 4 | 5 |
| 18. | Мне нравится чувствовать, как моя кожа касается кожи близкого мне человека. | 1 | 2 | 3 | 4 | 5 |
| 19. | В детстве, когда мы гуляли, родители часто брали меня за руку. | 1 | 2 | 3 | 4 | 5 |
| 20. | Почти каждый день я обнимаюсь и целуюсь. | 1 | 2 | 3 | 4 | 5 |
| 21. | Иногда мне хочется, чтобы меня обняли. | 1 | 2 | 3 | 4 | 5 |
| 22. | Мне нравится ощущение геля для душа на коже. | 1 | 2 | 3 | 4 | 5 |
| 23. | Мне приятно держаться за руки с человеком, который мне нравится. | 1 | 2 | 3 | 4 | 5 |
| 24. | Я часто целуюсь с любимым человеком. | 1 | 2 | 3 | 4 | 5 |
| 25. | В целом я могу описать себя как человека, который любит физические контакты. | 1 | 2 | 3 | 4 | 5 |
| 26. | Я считаю правильным обнять и погладить близкого человека, чтобы утешить его. | 1 | 2 | 3 | 4 | 5 |
| 27. | Мне нравится делать себе пилинг. | 1 | 2 | 3 | 4 | 5 |
| 28. | Поцелуи – приятный способ выразить свои романтические чувства. | 1 | 2 | 3 | 4 | 5 |
| 29. | Меня часто гладят. | 1 | 2 | 3 | 4 | 5 |
| 30. | Я часто держусь за руки с людьми, которые мне нравятся. | 1 | 2 | 3 | 4 | 5 |
| 31. | Мне нравится, когда к моей коже прикасаются другие люди. | 1 | 2 | 3 | 4 | 5 |
| 32. | Мне нравится поглаживать кожу любимого человека. | 1 | 2 | 3 | 4 | 5 |
| 33. | Я обнимаюсь при встрече со многими знакомыми. | 1 | 2 | 3 | 4 | 5 |
| 34. | Мне нравится принимать ванну с большим количеством пены. | 1 | 2 | 3 | 4 | 5 |
| 35. | Меня очень успокаивают объятия, когда я расстроен. | 1 | 2 | 3 | 4 | 5 |
| 36. | Мне нравится пользоваться косметическими масками для лица. | 1 | 2 | 3 | 4 | 5 |
| 37. | Мне нравится, когда мои друзья и родные обнимают меня при встрече. | 1 | 2 | 3 | 4 | 5 |

**Подсчет баллов.**

Большинство вопросов имеют прямое кодирование.

Совершенно не согласен  = 1, скорее не согласен = 2, не могу определиться = 3, скорее согласен = 4, совершенно согласен = 5.

Символ R рядом с номером вопроса означает, что для данного вопроса следует использовать обратное кодирование (совершенно не согласен  = 5, скорее не согласен = 4, не могу определиться = 3, скорее согласен = 2, совершенно согласен = 1).

Для подсчета баллов по каждой шкале опросника сложите баллы по следующим вопросам:

Отношение к социальным тактильным контактам (ОСТ): (*7 пунктов, минимальное значение 7, максимальное значение 35*): 1R, 11, 14, 25, 31, 33, 37

*Отношение к тактильным контактам с близкими людьми (ОТБ):* (*10 пунктов, минимальное значение 10, максимальное значение 50*): 9, 12, 15, 18, 21, 23, 26, 28, 32, 35

*Тактильные контакты в детстве (ТД)* (*8 пунктов, минимальное значение 8, максимальное значение 40*): 4, 5, 6, 8, 10, 16, 17, 19

*Отношение к уходу за кожей (ОТК)* (*7 пунктов, минимальное значение 7, максимальное значение 35*): 2, 3, 7, 22, 27, 34, 36

*Частота тактильных контактов с близкими (ЧТБ)* (*5 пунктов, минимальное значение 5, максимальное значение 25*): 13, 20, 24, 29, 30

Общая сумма баллов по всем шкалам может использоваться в качестве меры общей предрасположенности человека к социальным тактильным контактам (минимально возможное значение 37 баллов, максимально возможное значение 185 баллов).

**TEAQ-37 RUS**

Please select a response next to each of the statements below to indicate how much you agree or disagree with each statement.

|  |  | Disagree strongly | Disagree a little | Neither agree nor disagree | Agree a little | Agree strongly |
| --- | --- | --- | --- | --- | --- | --- |
| 1. | I dislike people being very physically affectionate towards me. (R) | 1 | 2 | 3 | 4 | 5 |
| 2. | I like using moisturisers on my skin. | 1 | 2 | 3 | 4 | 5 |
| 3. | I like using body lotions. | 1 | 2 | 3 | 4 | 5 |
| 4. | There was a lot of physical affection during my childhood. | 1 | 2 | 3 | 4 | 5 |
| 5. | My parents regularly cuddled me as a child. | 1 | 2 | 3 | 4 | 5 |
| 6. | As a child I would often hug family members. | 1 | 2 | 3 | 4 | 5 |
| 7. | I like to use bath essence when having a bath. | 1 | 2 | 3 | 4 | 5 |
| 8. | As a child I found a hug from my parents when I was upset made me feel much happier. | 1 | 2 | 3 | 4 | 5 |
| 9. | Kissing is a great way of expressing physical attraction. | 1 | 2 | 3 | 4 | 5 |
| 10. | As a child my parents would tuck me up in bed every night and give me a hug and a kiss goodnight. | 1 | 2 | 3 | 4 | 5 |
| 11. | Physical contact with other people is important to me. | 1 | 2 | 3 | 4 | 5 |
| 12. | Hugging someone is a good way of consoling them. | 1 | 2 | 3 | 4 | 5 |
| 13. | I can always find somebody to physically comfort me when I am upset. | 1 | 2 | 3 | 4 | 5 |
| 14. | I enjoy grooming other people’s skin. | 1 | 2 | 3 | 4 | 5 |
| 15. | I enjoy being cuddled by someone I am fond of. | 1 | 2 | 3 | 4 | 5 |
| 16. | My mother regularly bathed me as a child. | 1 | 2 | 3 | 4 | 5 |
| 17. | As a child my parents always comforted me when I was upset. | 1 | 2 | 3 | 4 | 5 |
| 18. | I enjoy the feeling of my skin against someone else’s if I know them intimately | 1 | 2 | 3 | 4 | 5 |
| 19. | As a child my parents would often hold my hand when I was walking along with them. | 1 | 2 | 3 | 4 | 5 |
| 20. | Most days I get a hug or a kiss. | 1 | 2 | 3 | 4 | 5 |
| 21. | Sometimes I just need to be hugged. | 1 | 2 | 3 | 4 | 5 |
| 22. | I like the feel of shower gels against my skin | 1 | 2 | 3 | 4 | 5 |
| 23. | I enjoy holding hands with someone I am fond of. | 1 | 2 | 3 | 4 | 5 |
| 24. | I often share a romantic kiss. | 1 | 2 | 3 | 4 | 5 |
| 25. | In general, I would describe myself as a physically affectionate person. | 1 | 2 | 3 | 4 | 5 |
| 26. | It’s good to console people you know well with strokes and hugs. | 1 | 2 | 3 | 4 | 5 |
| 27. | I like exfoliating my skin. | 1 | 2 | 3 | 4 | 5 |
| 28. | Kissing is an enjoyable part of expressing romantic feeling. | 1 | 2 | 3 | 4 | 5 |
| 29. | I often have my skin stroked. | 1 | 2 | 3 | 4 | 5 |
| 30. | I often hold hands with someone I am fond of. | 1 | 2 | 3 | 4 | 5 |
| 31. | I enjoy having my skin groomed by other people. | 1 | 2 | 3 | 4 | 5 |
| 32. | I like to stroke the skin of someone I know intimately. | 1 | 2 | 3 | 4 | 5 |
| 33. | I am on huggable terms with quite a few people. | 1 | 2 | 3 | 4 | 5 |
| 34. | I like having a bath with lots of bubble bath. | 1 | 2 | 3 | 4 | 5 |
| 35. | I find a hug very comforting when I am upset. | 1 | 2 | 3 | 4 | 5 |
| 36. | I like to use face masks on my skin | 1 | 2 | 3 | 4 | 5 |
| 37. | I like it when my friends and family greet me by giving me a hug. | 1 | 2 | 3 | 4 | 5 |

**Scoring**

Disagree strongly = 1, disagree a little = 2, neither agree nor disagree = 3, agree a little = 4, agree strongly = 5

R denotes items which are reverse scored (i.e. disagree strongly = 5, disagree a little = 4, neither agree nor disagree = 3, agree a little = 2, agree strongly = 1). Item numbers below indicate the items which belong to each of the subscales

Calculate the mean score for each subscale to obtain a subscale score:

Attitude to Friendly Touch subscale (AFT): 1R, 11, 14, 25, 31, 33, 37 (lowest obtainable score 7, highest obtainable score 35)

Attitude to Intimate Touch subscale (AIT): 9, 12, 15, 18, 21, 23, 26, 28, 32, 35 (lowest obtainable score 10, highest obtainable score 50)

Childhood Touch subscale (ChT): 4, 5, 6, 8, 10, 16, 17, 19 (lowest obtainable score 8, highest obtainable score 40)

Attitude to Self-Care subscale (ASC): 2, 3, 7, 22, 27, 34, 36 (lowest obtainable score 7, highest obtainable score 35)

Current Intimate Touch subscale (CIT): 13, 20, 24, 29, 30 (lowest obtainable score 5, highest obtainable score 25)

The total score may be calculated as the sum of the subscale scores and is considered to reflect a general predisposition towards social touch (lowest obtainable score 37, highest obtainable score 185)
