# Supplementary material for: Validation and psychometric properties of the Russian version of the Touch Experiences and Attitudes Questionnaire (TEAQ-37 Rus)
Source: PLoS One. 2018 Dec 13;13(12):e0206905. doi: 10.1371/journal.pone.0206905 (PMC6292699; doi:10.1371/journal.pone.0206905)
Supplement: S4 Table — (DOCX) [file pone.0206905.s004.docx]

Table 2.1. Factor structure of TEAQ-37 Rus (Stage 2 sample data).

Factor loadings, item-total correlations, and Cronbach's α if deleted are provided for all the items.

|  |  | AIT | ChT | ASC | AFT | CIT | Item-Total Corr. | Alpha if deleted |
| --- | --- | --- | --- | --- | --- | --- | --- | --- |
|  | I dislike people being very physically affectionate towards me. (R) | -0.083653 | 0.148654 | 0.147259 | 0.462789 | -0.161580 | 0.181151 | 0.921439 |
|  | I like using moisturisers on my skin. | 0.258026 | 0.194820 | 0.664562 | -0.160100 | 0.044691 | 0.460989 | 0.918240 |
|  | I like using body lotions. | -0.023252 | 0.184387 | 0.714086 | 0.099338 | 0.136080 | 0.455876 | 0.918326 |
|  | There was a lot of physical affection during my childhood. | 0.011399 | 0.762409 | 0.155586 | 0.195563 | 0.125259 | 0.515397 | 0.917480 |
|  | My parents regularly cuddled me as a child. | 0.048017 | 0.824418 | 0.047118 | 0.166562 | 0.102540 | 0.489643 | 0.917804 |
|  | As a child I would often hug family members. | 0.196689 | 0.713917 | 0.164026 | 0.108024 | 0.042167 | 0.529614 | 0.917292 |
|  | I like to use bath essence when having a bath. | 0.179911 | 0.107645 | 0.628363 | 0.189795 | -0.125901 | 0.431812 | 0.918597 |
|  | As a child I found a hug from my parents when I was upset made me feel much happier. | 0.168073 | 0.608284 | 0.225198 | 0.135389 | 0.107759 | 0.526293 | 0.917403 |
|  | Kissing is a great way of expressing physical attraction. | 0.519988 | 0.048173 | -0.072275 | 0.270110 | -0.046801 | 0.317671 | 0.919748 |
|  | As a child my parents would tuck me up in bed every night and give me a hug and a kiss goodnight. | 0.111131 | 0.628244 | 0.016132 | 0.007742 | -0.037439 | 0.319093 | 0.920038 |
|  | Physical contact with other people is important to me. | 0.293817 | 0.179719 | 0.255863 | 0.489790 | 0.158281 | 0.556108 | 0.917206 |
|  | Hugging someone is a good way of consoling them. | 0.635661 | 0.044733 | 0.237498 | 0.068498 | 0.062539 | 0.473445 | 0.918100 |
|  | I can always find somebody to physically comfort me when I am upset. | 0.209782 | 0.175531 | 0.100051 | 0.053069 | 0.622479 | 0.456770 | 0.918214 |
|  | I enjoy grooming other people’s skin. | 0.186383 | 0.233375 | 0.123968 | 0.692254 | 0.113279 | 0.517745 | 0.917496 |
|  | I enjoy being cuddled by someone I am fond of. | 0.714451 | 0.137794 | 0.002473 | 0.171912 | 0.038419 | 0.484503 | 0.918105 |
|  | My mother regularly bathed me as a child. | 0.084918 | 0.534343 | 0.194295 | -0.17336 | 0.190270 | 0.361775 | 0.919246 |
|  | As a child my parents always comforted me when I was upset. | -0.007601 | 0.732396 | 0.032887 | 0.016187 | 0.147843 | 0.374996 | 0.919136 |
|  | I enjoy the feeling of my skin against someone else’s if I know them intimately | 0.534729 | 0.068455 | -0.012071 | 0.373057 | 0.035346 | 0.419829 | 0.918640 |
|  | As a child my parents would often hold my hand when I was walking along with them. | 0.267404 | 0.512818 | 0.207528 | -0.179072 | 0.324792 | 0.490229 | 0.917827 |
|  | Most days I get a hug or a kiss. | 0.158318 | 0.190712 | 0.206679 | 0.066312 | 0.749078 | 0.527928 | 0.917320 |
|  | Sometimes I just need to be hugged. | 0.669022 | 0.074885 | 0.200187 | 0.037637 | 0.143907 | 0.509997 | 0.917632 |
|  | I like the feel of shower gels against my skin | 0.112740 | 0.111090 | 0.654654 | 0.266282 | -0.045103 | 0.463842 | 0.918119 |
|  | I enjoy holding hands with someone I am fond of. | 0.623569 | -0.062729 | 0.157752 | 0.181253 | 0.250141 | 0.491107 | 0.917987 |
|  | I often share a romantic kiss. | 0.380325 | 0.124148 | -0.041442 | 0.127634 | 0.594456 | 0.470794 | 0.918036 |
|  | In general, I would describe myself as a physically affectionate person. | 0.362636 | 0.123546 | 0.105707 | 0.586284 | 0.312939 | 0.595827 | 0.916698 |
|  | It’s good to console people you know well with strokes and hugs. | 0.700861 | 0.178921 | 0.037118 | -0.038850 | 0.108136 | 0.456167 | 0.918243 |
|  | I like exfoliating my skin. | -0.097576 | 0.033124 | 0.707076 | 0.175575 | 0.167032 | 0.387894 | 0.919098 |
|  | Kissing is an enjoyable part of expressing romantic feeling. | 0.724374 | 0.034835 | -0.086798 | 0.088129 | 0.143287 | 0.413915 | 0.918691 |
|  | I often have my skin stroked. | 0.078821 | 0.145116 | 0.149927 | 0.311221 | 0.752369 | 0.529286 | 0.917343 |
|  | I often hold hands with someone I am fond of. | 0.376937 | 0.023410 | 0.191894 | 0.457441 | 0.485777 | 0.610590 | 0.916353 |
|  | I enjoy having my skin groomed by other people. | 0.081681 | 0.030474 | 0.114856 | 0.743003 | 0.240709 | 0.433144 | 0.918460 |
|  | I like to stroke the skin of someone I know intimately. | 0.630061 | 0.084124 | -0.013925 | 0.231802 | 0.279529 | 0.520793 | 0.917526 |
|  | I am on huggable terms with quite a few people. | 0.100408 | 0.315827 | 0.173385 | 0.487047 | 0.126776 | 0.460739 | 0.918169 |
|  | I like having a bath with lots of bubble bath. | 0.232487 | 0.012436 | 0.614329 | 0.204904 | 0.059798 | 0.488266 | 0.917850 |
|  | I find a hug very comforting when I am upset. | 0.702993 | 0.104475 | 0.275341 | 0.020341 | 0.157809 | 0.575118 | 0.916824 |
|  | I like to use face masks on my skin | 0.053191 | 0.129080 | 0.789164 | 0.002854 | 0.286869 | 0.526732 | 0.917418 |
|  | I like it when my friends and family greet me by giving me a hug. | 0.501776 | 0.202040 | 0.372896 | 0.203692 | 0.124188 | 0.613296 | 0.916440 |
